# The biological applications of IPN hydrogels

**DOI:** 10.5599/admet.2398

**Published:** 2024-08-14

**Authors:** María I. León-Campos, Juan J. Mendoza, Hilda Aguayo-Morales, Luis E. Cobos-Puc, Denis A. Cabrera-Munguía, Jesús A. Claudio-Rizo

**Affiliations:** Facultad de Ciencias Químicas, Universidad Autónoma de Coahuila, Ing. J. Cárdenas Valdez S/N, República, 25280 Saltillo, Coahuila, México

**Keywords:** Bioactive gels, 3D bioprinting, tissue engineering, controlled drug release, regenerative medicine

## Abstract

**Background and purpose:**

Interpenetrating polymer network (IPN) hydrogels are an adaptable category of materials, exhibiting remarkable promise for various biological applications due to their distinctive structural and functional attributes. This review delves into the synthesis of IPN hydrogels through both physical and chemical methodologies, elucidating how these techniques allow for precise tailoring of mechanical properties, swelling behaviour, and biocompatibility.

**Experimental approach:**

We conducted an extensive literature review by searching well-established online research databases for articles published since 2009 to gather comprehensive data on IPN hydrogels.

**Key results:**

Our review highlights several critical applications of IPN hydrogels in the biomedical field; i) Tissue engineering: IPN hydrogels are evaluated for their capacity to emulate the extracellular matrix, making them excellent scaffolds for tissue engineering; ii) Controlled drug release: The ability of IPN hydrogels to modulate drug release rates and protect bioactive molecules is explored. Their structure enables sustained and targeted delivery of therapeutic agents, enhancing treatment efficacy; iii) 3D bioprinting: The use of IPN hydrogels as bioinks for 3D bioprinting is assessed, demonstrating their capability to construct intricate, biomimetic structures with high precision; and iv) Regenerative medicine: the development of biomimetic IPN hydrogels for regenerative medicine, emphasizing their potential to closely replicate natural biological environments, thereby promoting effective tissue repair and regeneration.

**Conclusion:**

IPN hydrogels emerge as a versatile and multifaceted platform with significant implications for advancing biomedical science and clinical therapies. Their diverse applications highlight their potential to revolutionize current biomedical practices and contribute to the development of innovative therapeutic solutions.

## Introduction

**Figure 1.**
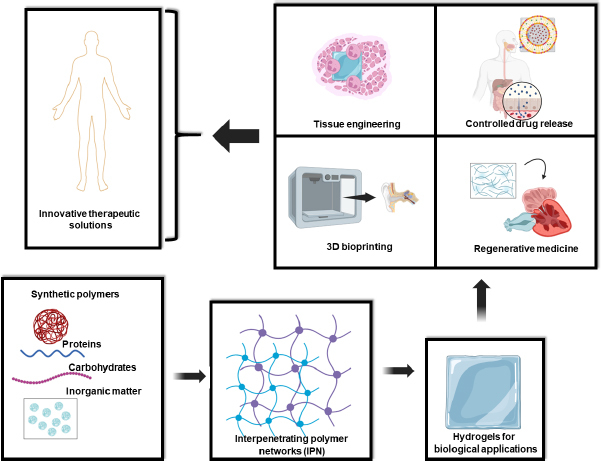
Preparation of IPN hydrogels with biopolymers, synthetic polymers, and inorganic materials for biological applications.

Interpenetrating polymer network (IPN) hydrogels represent a groundbreaking class of biomaterials, offering unparalleled versatility and functionality in various scientific and medical domains. These hydrogels consist of two or more polymer networks interlaced on a molecular scale but not covalently bonded [1-6]. This unique architecture endows IPN hydrogels with superior mechanical strength, resilience, and a remarkable ability to retain large volumes of water, making them ideal for a wide range of biological applications (Figure 1). The interwoven polymer networks within IPN hydrogels confer enhanced mechanical properties compared to single-network hydrogels. This structural synergy allows IPN hydrogels to withstand substantial deformation and stress, making them suitable for dynamic biological environments where they must maintain integrity under mechanical load [6-10]. IPN hydrogels exhibit exceptional swelling capacities, absorbing significant amounts of water without dissolving. This property is crucial for applications like tissue engineering and drug delivery, where the hydrogel must mimic the high-water content of biological tissues or control the release of therapeutic agents [1-2]. The biocompatibility of IPN hydrogels ensures minimal adverse reactions when in contact with biological tissues. Moreover, their properties can be finely tuned by adjusting the composition and crosslinking density of the polymer networks, allowing customization for specific applications [7-11]. IPN hydrogels can be designed to respond to various stimuli, such as pH, temperature, and ionic strength. This responsiveness enables the development of smart systems for controlled drug release and biomedical applications [11-13].

IPN hydrogels can be synthesized through diverse physical and chemical methods, each offering distinct advantages and pathways for tailoring their properties. In physical methods such as: i) sequential polymerization: In this approach, one polymer network is first formed, and the second network is subsequently polymerized within the pre-existing network. This method allows for the integration of polymers with varying properties, creating a robust interpenetrating structure [1-4], and ii) swelling equilibrium method: This technique involves swelling a pre-formed polymer network in a monomer solution, followed by polymerization of the second network within the swollen matrix. The resultant hydrogel benefits from the pre-existing stability of the network and the new additional functionalities of the network [13-18]. In chemical methods such as:

- simultaneous polymerization: Both polymer networks are formed concurrently from a mixture of monomers and crosslinking agents. This simultaneous formation results in a highly interpenetrated structure, providing uniformity and strong interaction between the networks [18-20], and
- sequential interpenetrating network formation: Like sequential polymerization, this method involves forming one network first, followed by the *in situ* polymerization of the second network. However, it emphasizes the chemical crosslinking of the second network while the first remains physically entangled, enhancing mechanical properties and structural integrity [20-24].

The distinctive properties and versatile synthesis methods of IPN hydrogels make them ideal candidates for numerous cutting-edge biomedical applications. IPN hydrogels are increasingly used as scaffolds that closely mimic the extracellular matrix (ECM), providing a conducive environment for cell growth and tissue regeneration. Their mechanical robustness and tunable properties allow them to support a wide variety of tissues, from soft cartilage to hard bone [1-2]. With their capacity to control drug diffusion rates and protect bioactive molecules, IPN hydrogels are at the forefront of developing advanced drug delivery systems. These hydrogels can be engineered to release therapeutic agents in a sustained and targeted manner, improving treatment outcomes and reducing side effects [24-26]. As bioinks, IPN hydrogels enable the precise 3D printing of complex biomimetic structures. This capability is essential for fabricating functional tissues and organs, paving the way for innovations in regenerative medicine and personalized medical treatments [26-30]. In regenerative medicine, IPN hydrogels serve as dynamic platforms that replicate natural biological environments, facilitating tissue repair and regeneration. Their ability to provide mechanical support and biochemical cues makes them ideal for regenerating damaged tissues and organs (Figure 1) [1,30-32].

In the dynamic field of biomaterials research, IPN hydrogels are emerging as a cornerstone technology. Their unique ability to interlace multiple polymer networks without covalent bonds enables a level of structural and functional control paramount for advanced therapeutic applications. The increasing focus on precisely controlling the structure and properties of IPN hydrogels reflects their potential to revolutionize a broad spectrum of biomedical solutions, from tissue engineering and controlled drug release to 3D bioprinting and regenerative medicine (Figure 1).

Central to the success of IPN hydrogels is the strategic selection and combination of chemical components. This choice directly influences the biofunctionality of the resulting material, dictating its performance in therapeutic contexts [20-38]. Biopolymers, such as proteins and carbohydrates, are often at the forefront of this selection process. Their intrinsic biocompatibility and ability to mimic the native ECM make them ideal candidates for creating scaffolds that promote cellular behaviours crucial for tissue regeneration-adhesion, migration, proliferation, and differentiation. Proteins like collagen and gelatin offer structural similarities to the ECM, providing a supportive framework that cells recognize and interact with naturally. Carbohydrates, including alginate and chitosan, contribute additional functionality, such as forming IPN hydrogels with specific mechanical properties and degradation rates suited to various biological environments [1-2].

To complement the natural attributes of biopolymers, synthetic polymers are often integrated into IPN hydrogels. These polymers can be precisely engineered to control the mechanical strength and degradation kinetics of the hydrogel, thereby extending its functionality and lifespan in biomedical applications. Synthetic components like poly(ethylene glycol) (PEG) or poly(lactic-co-glycolic acid) (PLGA) are commonly used to enhance the durability and tailor the biodegradation rates of IPN hydrogels. By adjusting the ratio and molecular weight of these synthetic polymers, researchers can create IPN hydrogels with bespoke physical properties suited to specific therapeutic needs. The integration of biopolymers with synthetic polymers through physical or chemical methods leads to the creation of hybrid IPN hydrogels. These methods allow for the functionalization of synthetic polymers within biopolymer frameworks, resulting in interpenetrated networks where the content of synthetic polymers directly influences key properties like swelling behaviour, mechanical resilience, and biodegradation. This precise control facilitates the design of hybrid IPN hydrogels with optimized performance for long-term biomedical applications, offering the robustness of synthetic materials combined with the biocompatibility of natural polymers (Figure 1) [15-25].

Adding another layer of complexity and functionality, various inorganic compounds, such as metallic nanoparticles and graphene derivatives or nanotubes, are being incorporated into IPN hydrogel matrices. These inclusions significantly alter the hydrophobic and hydrophilic interactions within the matrix, modulating tissue growth, cell differentiation, and material behaviour under therapeutic conditions. For instance, incorporating metallic nanoparticles can enhance the mechanical strength and electrical conductivity of the IPN hydrogel, which is beneficial for applications requiring robust and responsive materials. Graphene derivatives and nanotubes contribute to the control of the viscosity of matrix and hydrophilic interactions, essential for the formulation of advanced bioinks for 3D bioprinting. These modifications also allow for the encapsulation and controlled release of therapeutic molecules, providing sustained and targeted delivery that is crucial for effective treatments [10-22]. In the following sections of this review, we will delve into the fascinating world of IPN hydrogels and their growing relevance in modern biomedicine. We will begin with a detailed analysis of the materials and methods used for creating these hydrogels, exploring how combinations of different polymers and synthesis techniques enable the design of materials with unique and adjustable properties. Next, we will review the most recent and innovative applications of IPN hydrogels in therapeutic fields, highlighting their transformative potential in key areas such as tissue engineering, controlled drug release, regenerative medicine, and 3D bioprinting (Figure 1).

## Physical and chemical methods to obtain IPN hydrogels

IPN hydrogels represent a cutting-edge approach to addressing the inherent limitations of traditional polymers, including issues with reactivity, degradability, and processability. Unlike conventional copolymers or other multicomponent systems, IPN hydrogels are characterized by interweaving two or more polymer networks, which are held together through non-covalent interactions rather than covalent bonds. A defining feature of IPN hydrogels is that at least one polymer network is synthesized or crosslinked in the presence of another network. If only one of the polymer components is crosslinked, the structure is referred to as a semi-IPN. These unique structural characteristics endow IPN hydrogels with remarkable adaptability and make them suitable for diverse applications such as tissue engineering, drug delivery, 3D bioprinting, and regenerative medicine [1,2]. The formation of IPN hydrogels is influenced by the chemical composition and the synthesis route employed. IPN hydrogels can be classified based on their method of preparation, which can involve both covalent and non-covalent chemical bonds. Additionally, IPN hydrogels are categorized by their organization pattern into either sequential IPNs or simultaneous IPNs. These distinctions are largely dictated by the synthesis process, including the types of monomers used, the reaction mechanisms involved, and the crosslinking techniques applied. The versatile nature of IPN hydrogels, derived from their intricate synthesis and structural design, positions them as advanced multicomponent polymer systems with significant potential for innovative applications in various fields of science and technology [3,4]. The method used to obtain IPN hydrogels involves the interplay of covalent and non-covalent chemical bonds, which is influenced by the type of crosslinking employed. Crosslinking can be achieved through chemical means, physical means, or a combination of both [5,6].

### Covalent crosslinking: Enhancing stability and mechanical strength

Chemical covalent crosslinking is a key process that imparts stability and superior mechanical properties to IPN hydrogels. This process involves the formation of strong covalent bonds within and between polymer chains, creating a robust and permanent network. The resulting structures are highly durable and resistant to degradation over time, making them more stable compared to those formed through physical crosslinking methods. These diverse crosslinking strategies enable the tailoring of mechanical properties of IPN hydrogels and their stability, making them suitable for a wide range of biological applications [3,7,8].

### Chemical covalent crosslinking in IPN hydrogels: Techniques and advantages

Chemical covalent crosslinking in IPN hydrogels involves the formation of stable bonds between polymer chains, often facilitated by crosslinker agents (CAs). This process is crucial for creating durable and resilient hydrogel networks. Several methods are employed to achieve covalent crosslinking, each with its unique benefits and applications:

- Use of crosslinking agents: Small molecules such as glutaraldehyde, genipin, and formaldehyde are commonly used to form covalent bonds between polymer chains.
- Free radical polymerization: This is a widely used technique where monomers polymerize in the presence of a CA, forming a network of covalently bonded polymer chains. It allows for the creation of robust and stable hydrogel structures ideal for various applications.
- Click reactions: These reactions are highly efficient, occurring under simple conditions and offering specific and selective covalent bonding between polymer chains. Click reactions are known for their high yield, minimal by-products, and the ability to proceed under mild conditions, making them highly suitable for biomedical applications.
- Diels-Alder reaction: This method involves a single-step cycloaddition reaction between a diene and a dienophile, forming a network without the need for initiators or coupling agents. The Diels-Alder reaction is advantageous for its simplicity and the ability to form strong, thermally irreversible bonds.
- Schiff base formation: This process utilizes the reaction between amines and aldehydes or ketones to form imine linkages, integrating into the hydrogel network. Schiff base reactions are straightforward and efficient, creating hydrogels with tunable properties and responsiveness to environmental changes.
- Michael addition reaction: This nucleophilic addition reaction occurs between enones and nucleophiles, allowing for rapid covalent bonding. Michael addition reactions are characterized by their short reaction times and compatibility with biomolecules, making them ideal for biological and medical applications.
- Photo-activated crosslinking: In this method, crosslinking is initiated by exposure to UV light or high-energy radiation (such as gamma rays or electron beams), forming covalent bonds in the polymer network. This technique provides fast and spatially controlled network formation under mild conditions. Only irradiated areas undergo crosslinking, offering precision in hydrogel structuring.
- Enzyme-catalysed reactions: Polymers can be modified or used in conjunction with enzymes to facilitate covalent crosslinking. Enzyme-catalysed crosslinking offers high biocompatibility, control, and rapid gelation times. This method is particularly advantageous for applications requiring gentle conditions and strong, specific covalent bonds.

These diverse chemical crosslinking methods enable the customization of mechanical properties of IPN hydrogels and their functionality, making them highly versatile for a broad range of biological applications [3,7,8].

One of the primary challenges in covalent crosslinking is achieving a precise stoichiometric balance between reactive functional groups. Ideally, crosslinking agents should react completely with polymer chains to form a stable network. However, in practice, incomplete reactions often occur due to factors such as suboptimal reaction conditions or the presence of impurities. This can result in unreacted functional groups, which may lead to inconsistencies in the physical properties of hydrogel and hinder the formation of a well-defined network structure. Such incomplete crosslinking can compromise the mechanical strength and stability of the final product [3-4].

Covalent crosslinking involves multiple reactive sites, which can lead to competitive reactions that disrupt the desired crosslinking process. Simultaneous reactions, including side reactions or polymerization of unintended monomers, can interfere with the primary crosslinking pathway, affecting the uniformity and quality of the hydrogel. This complexity necessitates careful optimization of reaction conditions, such as temperature, pH, and concentration, to minimize the impact of these competing processes. The reaction conditions for covalent crosslinking, such as temperature and solvent type, can significantly influence the crosslinking efficiency and the final properties of the hydrogel [3-4]. Variations in these conditions can lead to inconsistent crosslinking, resulting in hydrogels with varying mechanical properties and swelling behaviours [4]. Moreover, certain crosslinking agents may be sensitive to environmental factors such as humidity or oxygen, which can further complicate the synthesis process and impact reproducibility. Some covalent crosslinking agents, particularly those that are highly reactive or involve toxic byproducts, can pose challenges related to Biocompatibility. The presence of residual crosslinking agents or their byproducts may affect the suitability of the hydrogel for biomedical applications. Ensuring these materials are biocompatible requires rigorous purification processes and thorough evaluation of potential cytotoxic effects, which can add complexity and cost to the hydrogel production process [3,7,8].

Scaling up the covalent crosslinking process from laboratory to industrial production presents additional challenges. The reaction conditions that work well on a small scale may not translate effectively to larger scales, leading to issues with consistency and yield. Process optimization at scale requires extensive trial and error to adjust parameters and maintain uniform quality, which can be resource-intensive and time-consuming.

Post-synthesis modifications of covalently crosslinked IPN hydrogels can be challenging due to the stability of the crosslinked network. While covalent crosslinking provides strong and stable networks, modifying these structures for specific applications or integrating additional functional groups can be difficult. This limitation requires innovative approaches to design crosslinked networks that are adaptable and versatile for various applications [7-8].

### Physical crosslinking in IPN hydrogels: Characteristics

While hydrogels with physical crosslinking generally exhibit weaker mechanical properties compared to their covalently crosslinked counterparts, they offer unique advantages due to the nature of non-covalent interactions. The bonds formed through physical crosslinking are typically less strong than covalent bonds, which results in hydrogels with lower mechanical stability. However, these non-covalent bonds provide significant benefits, particularly in terms of environmental responsiveness and reversibility. Physical crosslinking relies on various types of molecular interactions and secondary forces to form and stabilize the IPN hydrogels. Physical crosslinking mechanisms enable the design of IPN hydrogels with unique properties tailored for specific applications. The ability of these hydrogels to respond to environmental changes makes them particularly valuable for applications requiring adaptability and reversibility, such as in smart drug delivery systems, stimuli-responsive materials, and bioengineering scaffolds [3,8-10].

### Physical crosslinking mechanisms in IPN hydrogels

Physical crosslinking plays a crucial role in the formation of IPN hydrogels, offering a range of methods to create structures with unique properties. Unlike covalent crosslinking, which forms strong and permanent bonds, physical crosslinking relies on weaker, non-covalent interactions that can provide reversibility and environmental responsiveness. Here is a detailed look at the primary mechanisms involved in physical crosslinking of IPN hydrogels:

- Hydrogen bonding: Hydrogen bonds are formed between macromolecules containing polar elements, such as amino, hydroxyl, or carboxylic acid groups. These interactions occur when a hydrogen atom covalently bonded to an electronegative atom, like oxygen or nitrogen, is attracted to another electronegative atom with a lone pair of electrons. The formation and stability of hydrogen bonds in hydrogels are sensitive to changes in temperature and pH, making these hydrogels responsive to environmental conditions. Hydrogels formed through hydrogen bonding are useful in applications that require responsiveness to temperature or pH changes, such as drug delivery and biosensors.
- Ionic and electrostatic interactions: Physical crosslinking through ionic interactions involves the attraction between oppositely charged ions or functional groups within the polymer chains. Electrostatic interactions are particularly prevalent in polyelectrolyte-based hydrogels, where macromolecules with opposite charges or metal-ligand complexes interact to form stable structures. Commonly seen in hydrogels containing polymers with ionic groups, such as those used in superabsorbent materials or pH-sensitive hydrogels. These hydrogels are ideal for applications like controlled drug release and environmental sensing, where the ionic strength or pH can trigger changes in the hydrogel structure.
- Crystallization: Crystallization-based physical crosslinking occurs when segments of polymer chains align into ordered, crystalline regions. This process can be induced through thermal manipulation involving cycles of cooling or heating, which promotes the formation of crystalline domains that stabilize the hydrogel.
- Thermal manipulation: By carefully controlling the temperature, polymers can transition between sol and gel phases, leading to the entanglement and crystallization of polymer chains. This method is often used in hydrogels requiring enhanced mechanical strength and stability, such as in tissue engineering scaffolds or high-performance materials.
- Hydrophobic interactions: Hydrophobic interactions occur when non-polar segments of polymer chains aggregate to minimize their exposure to water. This process can be thermally induced or achieved through ultrasonic treatment, resulting in the formation of hydrogels through a sol-gel transition. Hydrophobic end groups in water-soluble polymers cluster together in aqueous environments, leading to network formation. Hydrogels formed via hydrophobic interactions are suited for applications that exploit their sol-gel transition properties, such as responsive drug delivery systems and smart materials.
- Host-Guest interactions: This strategy involves the inclusion of a guest molecule into the cavity of a host molecule, typically driven by non-covalent forces. Host molecules usually have large cavity volumes, while guest molecules are smaller and fit into the cavity of host. Host or guest molecules are integrated into the initial network during the formation of the IPN hydrogel, leveraging one of the previously mentioned mechanisms for stabilization. Hydrogels utilizing host-guest chemistry are valuable for applications requiring dynamic and reversible interactions, such as in self-healing materials and targeted drug-delivery systems.

These physical crosslinking methods offer versatile and adaptable pathways to tailor the properties of IPN hydrogels, enabling their use in a wide range of applications, from biomedical engineering to environmental technologies [3,8-10].

Physical crosslinking forms the network structure and relies on non-covalent interactions - such as hydrogen bonding, ionic interactions, hydrophobic forces, and van der Waals forces. While these interactions enable dynamic and stimuli-responsive behaviours, they also result in weaker and more transient crosslinks compared to covalent bonds. This can lead to mechanical instability, particularly under physiological conditions where external stresses or changes in environmental factors (e.g., temperature, pH) may disrupt the hydrogel network, compromising its structural integrity [3,8-10]. Achieving uniformity in the network structure is a significant challenge in physically crosslinked IPN hydrogels. The formation of physical crosslinks is often influenced by the rate of gelation, the concentration of polymers, and the presence of external stimuli. These factors can lead to a heterogeneous distribution of crosslinks, resulting in uneven mechanical properties and inconsistent swelling behaviour across the hydrogel. This variability can affect the reproducibility and reliability of the hydrogels in practical applications, particularly where precision is crucial [9]. Physically crosslinked hydrogels are highly sensitive to environmental changes, which can both be advantageous and a limitation. While this sensitivity enables responsive behaviour, such as controlled drug release or adaptive tissue scaffolding, it also makes the hydrogels susceptible to unintended degradation or loss of functionality. For instance, fluctuations in temperature or ionic strength can disrupt the physical crosslinks, leading to premature degradation or uncontrolled release of encapsulated agents. This instability poses challenges for the long-term storage and use of these hydrogels in real-world applications [7-8]. The reliance on weaker physical interactions for crosslinking often results in hydrogels with lower mechanical strength compared to their chemically crosslinked counterparts. This limitation restricts the use of physically crosslinked IPN hydrogels in applications where robust mechanical properties are essential, such as load-bearing tissue engineering or structural biomaterials. Enhancing the mechanical properties of these hydrogels without sacrificing their dynamic and responsive nature remains a key challenge [8-10]. The reversible nature of physical crosslinks allows for self-healing and adaptive behaviour, but it also introduces the challenge of maintaining structural permanence when required. In some applications, such as long-term implants or scaffolds for tissue engineering, the ability to maintain a stable structure over time is critical. The reversible crosslinks in physically crosslinked hydrogels may degrade or rearrange, leading to changes in shape, volume, or mechanical properties that could undermine their effectiveness in such contexts [8-10].

Designing physically crosslinked IPN hydrogels requires careful consideration of multiple factors, including polymer selection, crosslinking mechanism, and environmental triggers. The interplay between these elements can make the optimization process complex and time-consuming. Achieving the desired balance between responsiveness, mechanical strength, and stability often involves extensive experimentation, which can be resource-intensive and challenging to scale up for industrial production. While physical crosslinking avoids potentially toxic chemical agents, biocompatibility concerns are still associated with some physical crosslinkers, such as certain metal ions or synthetic polymers. Ensuring that the materials used in the hydrogel are biocompatible, non-immunogenic, and safe for long-term use in the body is essential, particularly for biomedical applications like drug delivery and tissue engineering. This requirement adds additional complexity to the design and synthesis of physically crosslinked IPN hydrogels.

### Enhancing IPN hydrogels: The synergy of chemical and physical crosslinking

Combining chemical and physical crosslinking methods in the development of IPN hydrogels results in materials with superior and balanced properties. This dual approach leverages the strengths of both crosslinking routes, creating hydrogels that are mechanically robust, adaptable, and functionally versatile. In the synthesis of IPN hydrogels, two primary structural configurations can be achieved depending on the crosslinking methods used:

- Semi-interpenetrating polymer network (Semi-IPN): In a semi-IPN, one polymer network is covalently crosslinked, while the second polymer remains physically entangled or loosely associated within the network. This structure allows the non-covalently bonded polymer to move and respond to environmental changes, imparting flexibility and dynamic responsiveness to the hydrogel. The covalently crosslinked network provides the necessary mechanical strength and stability.
- Full-Interpenetrating polymer network (Full-IPN): In a full-IPN, both polymer networks are covalently crosslinked in a more tightly interwoven structure. The dual covalent crosslinking creates a highly stable and durable hydrogel with enhanced mechanical properties. This configuration is particularly effective for applications requiring long-term structural integrity and high load-bearing capacity.

The synergy of chemical and physical crosslinking in IPN hydrogels leads to a global entanglement of polymer networks, which acts as a reinforcement mechanism [3,8]. This combined approach results in several key improvements:

- Enhanced mechanical strength: The integration of chemical crosslinks ensures robust and stable networks, while physical interactions contribute to the overall flexibility and toughness of the material.
- Improved physical properties: The dual crosslinking allows for tailored responsiveness to environmental stimuli, such as temperature, pH, and ionic strength, making the hydrogels suitable for a variety of conditions and applications.
- Superior drug loading and release: The complex network structure of IPNs can be optimized for high drug loading efficiency and controlled release, which is beneficial for drug delivery systems.
- Versatility in application: The ability to fine-tune the balance between covalent and physical crosslinks makes IPNs ideal for a wide range of applications, from biomedical to industrial uses.

Table 1 presents various examples of IPN and semi-IPN hydrogels formed through different crosslinking methods, highlighting the versatility and advantages of combining chemical and physical approaches. These examples would illustrate how specific combinations of crosslinking techniques can be employed to achieve desired properties and functionalities in IPN hydrogels.

**Table 1.** Examples of chemical and physical crosslinking methods in IPN and Semi-IPN hydrogel formations

Components	Type of network	Crosslinking method	Application	Refs.
γ-glutamic acid, lysine, and alginate	IPN	Physical	Scaffolds for osteochondral defect repair	[11]
PVA/CH	IPN	Physical	Bone repair materials	[12]
SF/collagen	IPN	Physical	Cell delivery and tissue engineering	[13]
CT/PVA/IT	Semi-IPN	Physical	Wound dressing	[14]
PGS-co-PEG-g-catechol	IPN	Physical	Wound dressing	[15]
P(SA-co-AAc))/PNIPAm	IPN	Physical	Artificial muscles	[16]
PEG/MeHA	IPN	Physical	Cartilage regeneration	[17]
Alginate-collagen-PU	IPN	Chemical	Biomedicine	[18]
PAAc-g-b-CD and PAAm	IPN	Chemical	Drug deliver system	[19]
PEG-Heparin/ PNIPAm	IPN	Chemical	Biomedical	[20]
PAAm, polyelectrolyte Na-Alg	Semi-IPN	Chemical	Wastewater treatment	[21]
Fibrin-alginate	IPN	Chemical	Cell delivery vehicles	[22]
CH/GelMA	Semi-IPN	Chemical	Tissue engineering	[23]
Pectin/Chitosan	Semi-IPN	Chemical	Drug delivery	[24]
PNIPAm/HA	IPN	Chemical	Drug deliver	[25]
PAAm/poly(acrylic acid) / PAAm	IPN	Chemical	Actuator	[26]
Glycol chitosan, dibenzaldhyde, PEG, Alginate	IPN	Chemical	Cell delivery	[27]
Collagen, HA	IPN	Chemical	Corneal regeneration	[28]
SBC/PDMAAm	IPN	Chemical-Physical	Tissue enginerring	[29]
SF-MeHA	IPN	Chemical-Physical	Tissue enginerring	[30]
Collagen-PEG-AgNPs	Semi-IPN	Chemical-Physical	Wound dressins	[31]
BC membrane, CT, glutaraldehyde	Semi-IPN	Chemical-Physical	Antibacterial applications	[32]
Collagen, PU, HEC, HPMC and Alm	Semi-IPN	Chemical-Physical	Wound dressing	[33]
HAPAM/PVA	IPN	Chemical-Physical	Biomedical	[34]
Polyvinyl alcohol (PVA)/PAAm	IPN	Chemical-Physical	Biomedicine	[35]
Gelatin/PAAm/clay	Semi-IPN	Chemical-Physical	Flexible sensor	[36]
Carboxymethyl chitosan/agarose	Semi-IPN	Chemical-Physical	Antibacterial	[37]
Collagen, PU, Zn-MOF	Semi-IPN	Chemical-Physical	Tissue engineering	[38]
Collagen, gum arabic, PU	Semi-IPN	Chemical-Physical	Antibacterial and Drug delivery	[39]

PVA/CH: Poly(vinyl) alcohol/Chitosan; SF: silk fibroin; IT: antibacterial agent, CT: chitin; PGS-co-PEG-g-catechol: poly(glycerol sebacate)-co-poly(ethylene glycol)-g-catechol; P(SA-co-AAc): poly(stearyl acrylate-co-acrylic acid, PNIPAm: poly(N-isopropylacrylamide); PEG: Poly(ethylene glycol), MeHA: methacrylated hyaluronic acid; PU: polyurethane; PAAc-g-β-CD: poly(acrylic acid)-graft-β-cyclodextrin; PAAm: polyacrylamide; GelMA: Gelatin methacryloyl; SBC: swim bladder collagen, PDMAAm: poly(N, N'-dimethylacrylamide); HAPAM: hydrophobically associated polyacrylamide; PU: polyurethane, HEC: hydroxyethylcellulose, HPMC: hydroxypropylmethylcellulose, Alm: starch; Zn-MOF: zinc-metal organic framework.

### Formation and structural requirements of IPN Hydrogels

The creation of IPN hydrogels involves specific structural requirements that determine their organizational pattern and functional capabilities. A variety of both natural and synthetic polymers can be employed to achieve the unique properties of IPN hydrogels. These polymers contribute different characteristics, offering flexibility in designing hydrogels for specific applications.

Types of polymers used in IPN hydrogels:

- Natural Polymers: Polysaccharides (*e.g.* alginate, chitosan, hyaluronic acid) and proteins (*e.g.* gelatin, collagen, silk fibroin). Natural polymers are known for their biocompatibility, biodegradability, and intrinsic biological activities. Due to their compatibility with living tissues and cells, they are often used in biomedical applications, such as tissue engineering and drug delivery.
- Synthetic Polymers: Hydrophilic polymers that contain functional groups like hydroxyl (-OH), carboxyl (-COOH), sulfonic acid (-SO_3_H), and amide (-CONH_2_). Synthetic polymers can be engineered to have precise mechanical properties, controlled degradation rates, and specific functional characteristics. This makes them highly versatile and suitable for a wide range of industrial and medical applications.

**Figure 2.**
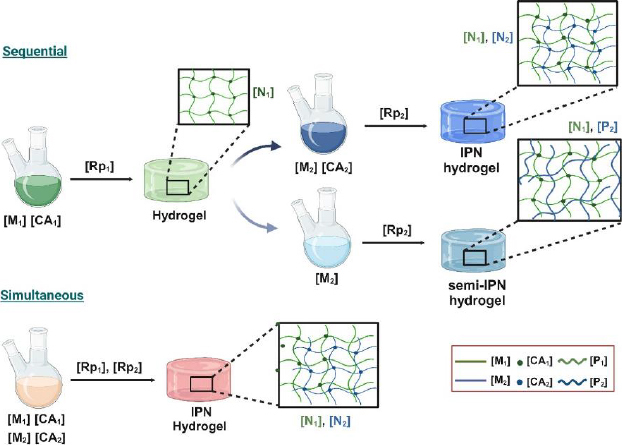
Illustration of the synthesis via sequential and simultaneous methods to produce either IPN or semi-IPN hydrogels. M_1_ and M_2_ represent monomers 1 and 2 (Note: It is also possible to start from the polymers instead monomers); CA_1_ and CA_2_ represent the crosslinker agents for each monomer (or polymer, P); Rp_1_ and Rp_2_ are the polymerization reaction involving the formation of P_1_ and P_2_ in the presence or absence of a crosslinker agent; finally, N_1_ and N_2_ represent the single-network hydrogels formed from each monomer (or polymer).

Combining polymers from these two groups makes it possible to tailor the properties of IPN hydrogels, resulting in materials that leverage the advantages of both natural and synthetic components (see Table 1). The combination of polymers from either group generates the possibility of obtaining IPN hydrogels [1,4,40]. As previously mentioned, the main preparation methods are sequential and simultaneous, producing either semi-IPN or IPN hydrogels. A representative illustration of both routes is shown in Figure 2.

The sequential IPN method is a multi-stage synthesis process. It initially involves the preparation of a first single-network hydrogel [N_1_] from a monomer [M_1_], which is then swollen into a solution containing another monomer [M_2_], an activator (crosslinking agent [CA_2_]) and an initiator, leading to *in situ* polymerization [Rp_2_] that creates a second network [N_2_], which interlaces with the first, resulting in an IPN hydrogel. If [CA_2_] is not present, a semi-IPN is formed, where a polymer formed (or added) is entrapped within [N_1_]. Therefore, many variations are possible and will depend mainly on the presence or absence of the crosslinking agents [CA_1_] and [CA_2_]. Another variation is the formation of a gradient IPN, characterized by the overall composition or crosslink density of the materials varying across the network, resulting in gradual changes in properties from the centre of the sample outward or from one location to another. In this variation of the sequential IPN method, the first single-network hydrogel [N_1_] is only partially swollen into the solution containing [M_2_] and [CA_2_], and its polymerization rate is faster than its diffusion rate into [N_1_], resulting in the formation of a gradient IPN [3,4,41]. Contrary to the sequential method, the simultaneous IPN method is a one-stage synthesis process. The components that will form both networks, monomers [M_1_ and M_2_], initiators, and crosslinker agents [CA_1_ and CA_2_], are added and mixed simultaneously, allowing polymerization reactions [Rp_1_ and Rp_2_] for each system to occur independently at the same time. It means noninterfering polymerization processes are necessary for each monomer to not interfere with one another; for instance, one network formation might proceed via a stepwise polymerization (polycondensation), while the other might occur via chain-growth reaction (free-radical polymerization). In this case, a semi-IPN is formed in the absence of [CA_1_] or [CA_2_]. This means that, either by sequential or simultaneous synthesis mechanisms, IPN or semi-IPN will be obtained by adding both crosslinkers or omitting one of them [41-43]. A general scheme describing both patterns of organization, sequential or simultaneous IPN, is shown in Figure 2.

Natural polymers are inherently complex, with their purity often influenced by the source and extraction process. For instance, collagen derived from animal tissues may contain residual proteins, lipids, or other biomolecules that can affect the consistency and reliability of the final hydrogel. Alginate, extracted from seaweed, may carry impurities like salts or polyphenols, which could interfere with crosslinking processes. The variability in the purity of natural polymers poses a challenge in achieving consistent hydrogel properties, particularly in applications requiring high precision, such as drug delivery or tissue engineering. In contrast, synthetic polymers like PMMA, PVA, and PEG are typically produced under controlled conditions, allowing for higher and more consistent purity. The controlled synthesis of these polymers enables precise tailoring of their molecular weight, functional groups, and polydispersity, resulting in more predictable and uniform behaviour in IPN hydrogel formation. The high purity of synthetic polymers ensures that the crosslinking reactions proceed as intended, minimizing the risk of unintended interactions or side reactions that could compromise the structural integrity of the hydrogel.

The inherent variability in natural polymers can make repeatability a significant challenge. The biological origin of these materials often leads to batch-to-batch differences in molecular weight, composition, and functional group availability. This variability can result in inconsistencies in the mechanical properties, swelling behaviour, and biodegradability of the resulting hydrogels. For example, the gelation behaviour of gelatine or fibrin can vary depending on the exact conditions under which they were extracted and processed, leading to difficulties in achieving consistent results across different batches. Synthetic polymers, with their controlled synthesis and well-defined chemical structures, offer superior repeatability. The ability to produce polymers with precise and consistent molecular characteristics ensures that IPN hydrogels synthesized using these materials exhibit uniform properties, batch after batch. This repeatability is particularly crucial in industrial applications, where large-scale production demands consistent quality. For instance, PEG, known for its biocompatibility and tunable properties, can be synthesized with a high degree of control, making it a reliable component in IPN hydrogel formulations where reproducibility is key.

The cost of natural polymers is influenced by their source, extraction, and purification processes. While some natural polymers like alginate are relatively inexpensive due to their abundant availability, others like collagen and fibrin can be costly, particularly when high purity is required. The need for extensive purification to remove contaminants and the variability in yield from natural sources add to the overall cost. Additionally, processing natural polymers often involves complex steps to ensure biocompatibility and functional integrity, further driving up costs. Synthetic polymers generally offer a more cost-effective solution, especially when large quantities are needed. The industrial-scale production of PMMA, PVA, and PEG benefits from economies of scale, leading to lower per-unit costs. Moreover, the consistent quality and high purity of synthetic polymers reduce the need for extensive post-processing, further lowering production costs. However, the initial investment in specialized equipment and raw materials for synthetic polymer production can be significant, particularly for high-performance or medical-grade polymers. Despite this, the long-term cost benefits of synthetic polymers in IPN hydrogel production are often substantial due to their repeatability and scalability.

In summary, IPN hydrogels are used as versatile multicomponent systems for various applications related to human health, especially in the pharmaceutical and biomedical fields, including drug delivery, tissue engineering, biomedical devices, 3D bioprinting and regenerative medicine. Combining physical and chemical methods to create IPN hydrogels using polymers with different properties complements one another, expanding their applicability. In general, research in physical and chemical methods to obtain IPN hydrogels focuses on exploring new crosslinking strategies, interpenetration routes, and synthesizing novel monomers for addressing emerging challenges.

## IPN hydrogels for tissue engineering

Tissue engineering integrates the natural ability of the body to regenerate with engineering principles to develop innovative solutions for repairing and replacing damaged tissues or organs. This cutting-edge field shows tremendous potential for advancing medical treatments and outcomes by cultivating living tissues. It aims to overcome the inherent limitations of conventional methods such as organ transplants (due to donor shortages and histocompatibility issues) and artificial prostheses (which provoke inflammatory responses and struggle to integrate with complex biological systems) [44]. The definition, originally proposed by Robert Langer and Joseph Vacanti in the early 1990s, has garnered widespread acceptance within the scientific and medical communities [45]. Despite exciting advancements in creating functional tissues and organs for transplantation, disease modelling, and drug testing, translating these successes from the laboratory to significant improvements in patient outcomes remains challenging. This gap underscores the complexity of turning tissue-engineered constructs into clinically viable solutions. Factors like scalability, integration with host tissues, immune response, and long-term functionality are crucial considerations in bridging this divide. Addressing these challenges is essential for realizing the full potential of tissue engineering in clinical practice [46].

**Figure 3.**
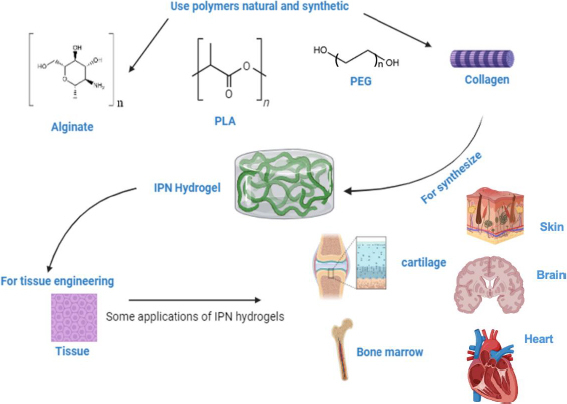
Use of IPN hydrogels based on natural and synthetic polymers in tissue engineering strategies.

IPN hydrogels have gained significant traction in tissue engineering due to their ability to mimic the ECM of the body, exhibiting similar mechanical and biochemical properties. They find wide application in bone tissue scaffolds, contact lenses, wound healing dressings, and hygiene products [47]. IPN hydrogels stand out for their unique structure formed by interpenetrating two or more crosslinked polymer networks. This configuration allows IPN hydrogels to synergistically combine the superior properties of each polymer component, resulting in enhanced mechanical strength, durability, and biological functionality [48]. IPN hydrogels are highly versatile and have demonstrated immense potential in tissue engineering. Their ability to mimic the mechanical properties of native tissues, along with their bionic adaptability, viscoelasticity, and tunable properties, makes them particularly suitable for various approaches to tissue engineering [48]. Furthermore, IPN hydrogels contribute to improved biocompatibility, cell adhesion, and tissue integration, further solidifying their role as promising candidates for creating advanced tissue scaffolds [5]. Figure 3 illustrates the use of natural and synthetic polymers in synthesizing IPN hydrogels for various tissue engineering applications. Hydrogel scaffolds with an IPN structure offer significant advantages over single-component gels, particularly in replicating the structure and properties of the native ECM found in cartilage tissue [49]. It is important to note that IPN hydrogel systems can be classified based on their composition, with natural polymers such as polysaccharides (*e.g.* alginate, starch, cellulose), proteins (*e.g*., collagen), and animal derivatives (*e.g.* chitosan, hyaluronic acid, pectin) being particularly noteworthy. These natural polymers possess several advantageous properties that render them highly suitable for tissue engineering. Most natural polymers can form hydrogels, which are colloids composed of long polymeric chains with a high-water content (up to 99 %). This characteristic makes them an ideal class of material for creating bioinspired scaffolds due to their strong resemblance to the ECM [50]. Incorporating synthetic polymers into these natural hydrogels often results in a significant enhancement in strength and stability [50]. This hybrid approach combines the beneficial properties of natural materials with the enhanced mechanical characteristics provided by synthetic components, making them ideal candidates for applications requiring robust and biocompatible scaffolds in tissue engineering.

IPN hydrogels of diverse compositions have been prepared for various strategies in tissue engineering taking advantage of its structural characteristics and physicochemical properties:

- Soft tissue engineering: The properties of IPN hydrogels are engineered to mimic the natural environment of soft tissues while offering mechanical support and promoting cellular activities essential for tissue regeneration. Here are some key properties of IPN hydrogels for soft tissue engineering: i) Biocompatibility: IPN hydrogels are designed to be biocompatible, meaning they are well-tolerated by biological tissues without causing adverse reactions. This property is crucial for applications where the hydrogel interacts closely with living cells and tissues; ii) Water absorption and retention: IPN hydrogels are primarily composed of water, allowing them to mimic the natural hydration state of soft tissues. IPN hydrogels have high water absorption capacities and can retain water within their structure, providing a moist environment that supports cell survival and growth; iii) Mechanical properties: Tailoring the mechanical properties of IPN hydrogels is essential to match those of the target soft tissues. These hydrogels can be engineered to have tunable elasticity, stiffness, and strength, which are critical for providing structural support and maintaining shape integrity within the dynamic environment of the body; iv) Porosity and permeability: The porous structure of IPN hydrogels can be controlled to resemble the native ECM of soft tissues. This porosity allows for nutrient and waste exchange, as well as cell migration throughout the hydrogel matrix, facilitating tissue integration and regeneration; v) Biodegradability: Depending on the application, IPN hydrogels can be designed to degrade over time through enzymatic or hydrolytic processes. Controlled degradation is advantageous as it allows for gradual replacement of the hydrogel by newly formed tissue, promoting long-term tissue regeneration and integration; vi) Bioactive functionalization: Surface modification or incorporation of bioactive molecules (such as growth factors, peptides, or ECM components) into IPN hydrogels can enhance their bioactivity. These bioactive cues can promote specific cellular behaviours such as adhesion, proliferation, differentiation, and tissue-specific ECM production; vii) Ease of fabrication and customization: IPN hydrogels can be synthesized using a variety of methods, including chemical crosslinking, physical gelation, and 3D printing techniques. This versatility allows for precise control over hydrogel composition, structure, and properties, enabling customization based on specific tissue engineering requirements, and viii) Sterilizability: Hydrogels intended for clinical applications must be sterilizable without compromising their physical or biochemical properties. IPN hydrogels can often be sterilized using methods such as gamma irradiation, ethylene oxide gas, or autoclaving, ensuring safety and sterility for *in vivo* applications [43-53].
- Hard tissue engineering: IPN hydrogels possess several advantageous properties that make them promising candidates for hard tissue engineering applications. Hard tissues, such as bone and cartilage, require materials that can mimic their mechanical properties and provide a supportive environment for cellular growth and differentiation. Here are some key properties of IPN hydrogels relevant to hard tissue engineering: i) Mechanical strength and stability: IPN hydrogels can be engineered to have robust mechanical properties, including stiffness and strength, which are crucial for supporting hard tissues like bone. These hydrogels can withstand mechanical stresses and maintain structural integrity over time, providing a scaffold that mimics the natural environment of hard tissues; ii) Biocompatibility: Like in soft tissue engineering, biocompatibility is essential for hard tissue applications. IPN hydrogels are designed to be biocompatible, ensuring they do not elicit adverse immune responses and can support cell adhesion, proliferation, and differentiation necessary for tissue regeneration; iii) Bioactivity and osteoinductivity: IPN hydrogels can be functionalized with bioactive molecules, such as growth factors or peptides, that promote osteogenesis (bone formation) and stimulate bone cell activity. This bioactivity enhances the ability of IPN hydrogel to guide and accelerate the regeneration of hard tissues; iv) Porosity and permeability: The porous structure of IPN hydrogels can be tailored to resemble the natural extracellular matrix of hard tissues. This porosity facilitates nutrient diffusion, waste removal, and cell infiltration throughout the hydrogel matrix, promoting tissue integration and new bone formation, and v) Biodegradability: Controlled degradation of IPN hydrogels is advantageous for hard tissue engineering, as it allows gradual replacement of the scaffold by newly formed bone tissue. Degradable IPN hydrogels ensure that the engineered construct evolves into natural tissue without the need for surgical removal, supporting long-term tissue regeneration [53-60].
- Cardiac tissue engineering: IPN hydrogels are biocompatible and well-tolerated by cardiac cells and tissues. This property is essential for creating a supportive environment that does not elicit adverse immune responses, allowing cell adhesion, proliferation, and differentiation. The mechanical properties of IPN hydrogels can be tuned to match those of native cardiac tissue, which is crucial for providing structural support. These IPN hydrogels can mimic the elasticity and stiffness required to withstand the dynamic mechanical forces of the heart. Some IPN hydrogels can be engineered to possess electrical conductivity or be functionalized with conductive materials. This property is beneficial for cardiac tissue engineering as it supports the propagation of electrical signals essential for synchronized contraction (heart beating). IPN hydrogels can be modified to incorporate bioactive molecules, such as growth factors or peptides, that promote angiogenesis (formation of new blood vessels) and cardiac cell maturation. This bioactivity enhances the ability of IPN hydrogel to support tissue regeneration and improve cardiac function. Controlled degradation of IPN hydrogels allows for gradual replacement by new cardiac tissue. This process supports integration with the surrounding tissue and facilitates long-term functional recovery. IPN hydrogels can be fabricated using methods compatible with cardiac tissue engineering, such as photopolymerization or crosslinking techniques. This versatility enables the creation of complex structures and customized scaffolds tailored to specific patient needs [3,10,55-60].
- Nerve tissue engineering: Like cardiac tissue engineering, IPN hydrogels are biocompatible with nerve cells and tissues. They provide a supportive matrix that promotes cell survival and growth without inducing inflammation or toxicity. IPN hydrogels can be designed to have mechanical properties that match those of nerve tissue, including elasticity and flexibility. These properties are crucial for creating a scaffold that can guide nerve regeneration and withstand mechanical stresses. The porous structure of IPN hydrogels supports nutrient diffusion and waste removal, facilitating cell infiltration and tissue integration. This feature is essential for nerve tissue engineering, promoting axonal growth and functional recovery. IPN hydrogels can be functionalized with bioactive molecules, such as neurotrophic factors or ECM components, to enhance nerve cell adhesion, neurite outgrowth, and synapse formation. This bioactivity supports the regeneration of damaged nerves and enhances functional recovery. The electrical conductivity is beneficial for nerve tissue engineering as it supports electrical signalling between neurons and promotes neural network formation. Controlled degradation of IPN hydrogels allows for gradual replacement by new nerve tissue, supporting integration and functional recovery. Degradable IPN hydrogels ensure that the engineered scaffold evolves into natural tissue without the need for surgical removal [1,15,55-60].

Table 2 presents studies highlighting IPN hydrogels that demonstrate applications in tissue engineering.

**Table 2.** Some studies in which IPN hydrogels have been synthesized for tissue engineering.

Composition	Main characteristic	Tissue engineering application	Ref.
Collagen Glycosaminoglycans (GAGs)	Good cytocompatibility. IPN hydrogels upregulate cartilage-specific gene expression and promote chondrocytes to secrete glycosaminoglycan and collagen II	Promising hydrogel scaffolds for cartilage tissue engineering	[53]
Methacrylate Gelatin Silk fibroin	Lower swelling ratio, higher compressive modulus, and slower degradation rate	Suitable for various tissue engineering and regenerative medicine, particularly in soft tissues	[54]
Alginate Hydroxyethyl-methacrylate-derivatized dextran	IPN beads support good cell survival and differentiation of equine chondrocytes	Injectable *in situ* forming hydrogels for protein delivery and hard and soft tissue engineering	[55]
Hyaluronic acid Silk fibroin (SF)	High SF concentrations enhance viscoelastic modulus; IPN hydrogels promote differentiation of mesenchymal stem cells into nucleus pulposus (NP) cells	Bone tissue engineering strategies	[56]
Poly(ethylene glycol) diacrylate (PEG-DA) Agarose	Improved elastic modulus	Nerve, cardiac and cartilage tissue engineering	[57]

## Biomimetic IPN hydrogels for regenerative medicine

Regenerative medicine stands at the frontier of modern healthcare, driven by the profound goal of repairing or replacing damaged tissues and organs. This emerging discipline harnesses the power of stem cells, growth factors, and biomaterials to foster the regeneration of body parts compromised by disease, injury, or congenital conditions. Among these biomaterials, IPN hydrogels have emerged as some of the most promising candidates, offering unparalleled versatility and functionality in regenerative medicine applications. IPN hydrogels are fascinating materials composed predominantly of water, held together by a network of polymer chains. This unique IPN structure endows hydrogels with a remarkable combination of elasticity and strength. These properties are crucial because they allow IPN hydrogels to replicate the mechanical characteristics of various tissues, from the pliable consistency of cartilage to the firm resilience of the myocardium. This mimicry is vital, ensuring that the IPN hydrogels can endure the dynamic loads and stresses that living tissues encounter daily [61].

Beyond their mechanical properties, IPN hydrogels can be intricately designed to emulate the natural tissue microenvironment. This includes presenting biochemical signals essential for cell adhesion, proliferation, and differentiation. Such cues are crucial for the successful integration and functionality of regenerated tissues. By carefully selecting and engineering the constituent polymers at a molecular level, scientists can create IPN hydrogels that provide the necessary support and signals for cellular activities while being biocompatible and immuno-tolerant. The selection of polymers for IPN hydrogels is a meticulous process aimed at balancing biocompatibility, biodegradability, and functional performance. Natural polymers like collagen and chitosan are often chosen for their inherent biological compatibility and ability to support cell growth. Collagen, a major component of the ECM, provides structural integrity and promotes cellular interactions, making it an excellent choice for hydrogel composition. Chitosan, derived from chitin, offers antimicrobial properties and facilitates wound healing, adding another layer of functionality to the hydrogel. On the other hand, synthetic polymers like polyethylene glycol (PEG) and polyacrylamide bring different advantages. PEG is widely appreciated for its non-immunogenic and highly tunable nature, allowing for precise control over the physical and chemical properties of IPN hydrogel. Polyacrylamide, known for its robustness and stability, can be engineered to provide mechanical support in environments where high durability is required.

### Mimicking the microenvironment

To ensure the IPN hydrogels effectively support tissue regeneration, they must closely mimic the natural microenvironment of the tissues intended to replace or repair. This involves replicating the physical structure and mechanical properties of the tissues and incorporating biochemical signals that drive cellular processes. For instance, in cardiac tissue engineering, IPN hydrogels can be infused with growth factors that promote angiogenesis (the formation of new blood vessels) and the proliferation of cardiomyocytes (heart muscle cells). This creates a conducive environment for the growth and integration of new cardiac tissue, which is essential for repairing damaged hearts. Similarly, for nerve tissue engineering, hydrogels can be functionalized with neurotrophic factors that encourage neuron growth and axonal extension, which are critical for nerve repair and regeneration. An important feature of IPN hydrogels in regenerative medicine is their ability to be gradually absorbed by the body as natural tissue regenerates. This biodegradability is carefully controlled to ensure that the hydrogel provides support only as long as necessary and then seamlessly transitions to allow the new tissue to take over. This process is engineered at the molecular level, selecting polymers and crosslinking methods that degrade at a rate matching the tissue regeneration pace. By fine-tuning these properties, scientists can create IPN hydrogels that support tissue regeneration and promote the integration of the newly formed tissue with the surrounding native tissue. This seamless integration is crucial for restoring full functionality to the regenerated tissue and for the long-term success of regenerative therapies.

### Versatility of IPN hydrogels in regenerative medicine

IPN hydrogels are emerging as a cornerstone in the realm of medical applications thanks to their remarkable properties, such as biocompatibility and the ability to be finely tuned for specific purposes. This unique structure endows them with a combination of properties from the individual polymers, leading to enhanced functionality and performance. One of the most compelling aspects of IPN hydrogels is their functionalization versatility, allowing them to be tailored for a wide range of regenerative medicine applications. This adaptability opens up exciting possibilities for advancing bone regeneration, skin healing, and nerve repair treatments, among others.

- IPN hydrogels in bone regeneration: IPN hydrogels are increasingly used as scaffolding materials for bone regeneration. Their mechanical strength and structural integrity provide the necessary support to withstand the stresses encountered in bone tissue. Moreover, these hydrogels can be engineered to release growth factors in a controlled manner, which is critical for stimulating new bone formation and promoting the healing process [62]. The incorporation of bioactive nanoparticles into IPN hydrogels further enhances their functionality. These nanoparticles can be designed to mimic the mineral content of bone, providing additional cues that promote osteogenesis (the formation of new bone tissue). For example, calcium phosphate nanoparticles can be integrated into the hydrogel matrix to create a microenvironment conducive to bone growth and mineralization. This combination of structural support and bioactivity makes IPN hydrogels a powerful tool for treating bone defects and fractures.
- IPN hydrogels in skin regeneration: In the field of skin regeneration, IPN hydrogels offer a dynamic solution for healing and repair. Their ability to retain moisture and provide a protective barrier makes them ideal for use in wound dressings and skin grafts. Additionally, these hydrogels can be functionalized to serve as delivery matrices for growth factors and stem cells, which are essential for promoting tissue repair and regeneration [63]. Growth factors like epidermal growth factor (EGF) or fibroblast growth factor (FGF) can be encapsulated within the hydrogel and released over time to accelerate the healing process. Meanwhile, stem cells embedded in the hydrogel can differentiate into various skin cell types, contributing to the reconstruction of damaged tissue. This controlled release and cellular support help to improve wound healing outcomes, reduce scarring, and restore the natural function of skin and appearance.
- IPN hydrogels in nerve repair: Nerve repair presents unique challenges due to the complex and delicate nature of neural tissues. IPN hydrogels offer a promising approach by providing a favourable environment for axonal regeneration and the reconstruction of neuronal connections [64]. Their tunable properties allow for the creation of scaffolds that support the growth and guidance of nerve cells, promoting the restoration of nerve function after injury. These IPN hydrogels can be functionalized with neurotrophic factors, which are proteins that support the growth, survival, and differentiation of neurons. By incorporating such factors, IPN hydrogels can enhance the regenerative capacity of the nervous system, facilitating the repair of damaged nerves and improving the recovery of sensory and motor functions. Moreover, the mechanical properties of IPN hydrogels can be adjusted to match the softness and flexibility of nerve tissues, providing the necessary support while minimizing damage and irritation to the delicate nerve fibres. This adaptability is crucial for creating scaffolds that not only support but also integrate seamlessly with the existing neural network.

Table 3 provides a snapshot of the diverse applications of IPN hydrogels leveraging the biomimetic characteristics of IPN hydrogels for applications in regenerative medicine.

**Table 3.** Some examples of biomimetic IPN hydrogels with great potential in regenerative medicine.

Composition	Synthesis	Crosslinker	Application	Ref.
Mg-doped Hydroxyapatite/Collagen (COL)	Acid/Base	Ribose	Bone regeneration	[65]
Hyaluronic acid (HA) / Gelatin (GEL) conjugated with phenolic groups	Enzymatic	Horseradish peroxidase/H_2_O_2_	Cartilage regeneration	[66]
HA methacrylate/GEL	Photopolymerization	Eosin Y/triethanolamine/ /white light	Corneal regeneration	[67]
Cyclodextrin-GEL/Adamantane-modified HA	Hydration	-	Skin regeneration	[68]
HA methacryloyl/Poly(N-hydroxyethyl acrylamide)	Photopolymerization	I 2959/UV light	Wound healing	[69]
HA/Alginate (ALG)/Polyvinyl alcohol loaded with cefotaxime (CTX)	Solvent cast	Glycerol	Release of CTX in skin wounds	[70]
GEL methacryloyl/Poly ethylene oxide loading with decalcified bone matrix, poly (lactic-co-glycolic acid), and vascular endothelial growth factor	Photopolymerization	Lithium phenyl-2,4,6-trimethylbenzoyl-phosphinate (LAP)/UV light	Vascularized bone	[71]
HA acrylate	Michael reaction	Matrix metalloproteinase (MMP)	Myocardial regeneration	[72]
Methacrylamide functionalized COL/chondroidtin sulphate-methacrylate	Gelation	Ammonium persulphate/ /tetramethylethylenediamine /20°C	Kidney regeneration	[73]
Sodium ALG/Graphene	Hydration	Calcium chloride	Nerve regeneration	[74]
Silk fibroin	Enzymatic	Horseradish peroxidase/ / H_2_O_2_	Tumour growth suppression	[75]
GEL methacrylate/ALG modified with dopamine loaded with adipose-derived mesenchymal stem cells exosomes	Photopolymerization	LAP/UV light	Liver regeneration	[76]
Polyethylene glycol (PEG)/Hydroxyapatite (HA) loaded with RGD peptide, bone morphogenetic protein 2 (BMP-2), and bone marrow stromal cells (BMSCs)	Enzymatic	Transglutaminase factor XIIIa	Bone marrow organoids	[77]

### Leveraging IPN hydrogels for the creation of artificial organoids in regenerative medicine

**Figure 4.**
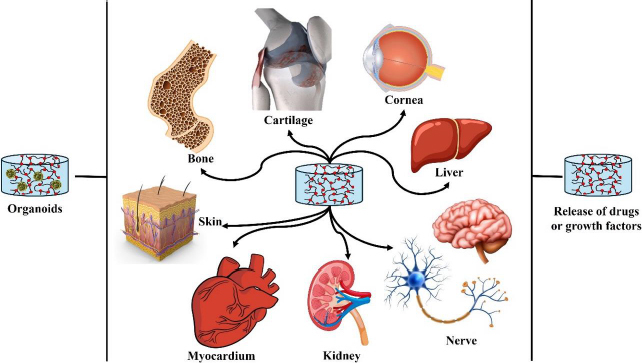
IPN hydrogels have been tested for a variety of applications, including the regeneration of tissues such as bone, skin, heart muscle, kidney, liver, nerve and cornea.

The field of regenerative medicine is rapidly advancing, with a particular focus on developing innovative solutions to address organ failure. Recently, researchers have turned their attention to the use of IPN hydrogels to create artificial organoids, such as bone marrow, kidneys, and hearts. These organoids are designed to replace the vital functions of failing organs, offering new hope for patients suffering from chronic conditions or acute damage that compromises organ function [78]. Organoids are three-dimensional (3D), *in vitro* models of organs that closely mimic the structure and functionality of their natural counterparts. They provide a highly controlled environment where cells can grow, differentiate, and organize into complex tissues that resemble real organs. This ability to recreate the architecture and function of organs on a miniature scale makes organoids invaluable tools for studying disease mechanisms, testing drug responses, and potentially serving as functional replacements for damaged tissues in therapeutic applications. IPN hydrogels have emerged as a key player in the development of artificial organoids due to their superior ability to mimic the ECM. The ECM is a crucial component of the tissue microenvironment, providing structural support and biochemical signals that guide cell behaviour. IPN hydrogels, with their intricate network of interwoven polymer chains, can replicate the physical and biochemical properties of the ECM more closely than other materials. IPN hydrogels can be engineered to provide the precise biochemical and mechanical signals necessary for cell differentiation and organization. These signals are critical for the development of organoids, as they guide the cells to form structures and functions like those of natural tissues. For example, specific growth factors and adhesion peptides can be incorporated into the hydrogel matrix to promote cell attachment, proliferation, and differentiation, driving the formation of organoid structures that accurately reflect the targeted organ. One of the significant advantages of IPN hydrogels is their ability to allow uniform cell distribution within the scaffold. This uniformity is essential for the formation of complex 3D structures that are critical for organoid functionality. Unlike traditional two-dimensional cultures, IPN hydrogels support the spatial organization of cells in three dimensions, facilitating the development of organoids with intricate architectures and functional layers that mimic real organs. IPN hydrogels can be customized to incorporate a wide range of bioactive components that enhance their functionality. Growth factors, which are proteins that regulate cell growth and differentiation, can be embedded within the hydrogel to create a nurturing environment that supports the development and maturation of organoid tissues. Additionally, adhesion peptides can be added to promote stronger cell-hydrogel interactions, further enhancing the stability and functionality of the growing organoid (Figure 4). The recent applications in artificial organoids encompass:

- Bone marrow organoids: Bone marrow is a highly specialized tissue responsible for producing blood cells. Creating bone marrow organoids using IPN hydrogels mimics the bone marrow niche's complex, supportive ECM. These organoids can provide a platform for studying hematopoiesis (blood cell formation) and testing treatments for blood disorders. IPN hydrogels facilitate the replication of the bone's unique microenvironment of marrow, enabling the development of functional bone marrow tissues *in vitro* [77].
- Kidney organoids: Kidney organoids aim to replicate the intricate filtration structures and cellular organization of the kidney. IPN hydrogels support the formation of nephron-like structures, which are essential for mimicking kidney function. By providing the right biochemical cues and mechanical support, IPN hydrogels help in developing kidney organoids that can be used to study kidney diseases and potentially serve as functional units for kidney repair [73].
- Heart organoids: Heart organoids, or cardiac organoids, require precise mimicking of the mechanical and electrical properties of the heart. IPN hydrogels can be engineered to provide the necessary support for cardiomyocytes (heart muscle cells) to organize into contracting tissues. These organoids can be used to model cardiac function, study heart diseases, and develop new treatments for cardiac conditions. The ability of IPN hydrogels to promote cell alignment and synchronized contraction is crucial for creating functional heart organoids [72].

The IPN hydrogels can also be designed to deliver drugs in a controlled and sustained manner, improving therapeutic efficacy and reducing side effects [79]. IPN hydrogels can release drugs by diffusion into the circulating environment, by controlled degradation of the drug-releasing hydrogel, and in response to stimuli such as changes in pH, temperature, or the presence of certain biomolecules. Some of their advantages are that: i) they allow prolonged drug release, improving therapeutic efficacy and reducing the frequency of administration; they can encapsulate large quantities of drugs, both hydrophilic and hydrophobic; ii) they can be designed to release drugs at specific rates, adapting to different therapeutic needs; and iii) they protect the encapsulated drugs from premature degradation before reaching the site of action [80]. The greatest potential for this type of IPN hydrogel is in chemotherapy, antibiotic therapy and regenerative medicine. However, the use of IPN hydrogels for drug delivery faces certain challenges, such as their production, which can be low yield and high cost. Another point to consider is the stability of the drug, as some may degrade or lose activity during the encapsulation or release process or simply because they are in an aqueous medium. In this sense, this field has no regulations, so their safe use is not yet guaranteed. The translation of biomimetic IPN hydrogels into the clinic is promising, but their application is still in the distant future, aspects such as the reproducibility and scalability of their production, full understanding of the interactions between the material and the host tissue, and strict regulation for their approval are significant barriers. However, continued advances in materials science and biomedical engineering promise to overcome these hurdles. The future of biomimetic IPN hydrogels in regenerative medicine is bright. The integration of emerging technologies such as 3D printing and synthetic biology will enable the design of customized and more sophisticated hydrogels. These developments will improve clinical outcomes and expand the scope of regenerative medicine to treat a wider variety of medical conditions.

## IPN hydrogels for controlled drug release

**Figure 5.**
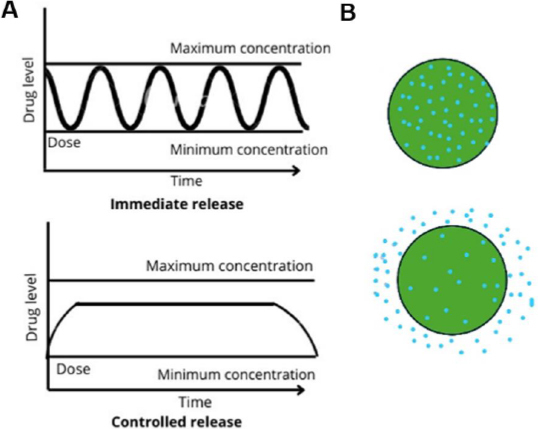
a) Immediate and controlled release curve of drugs and b) matrix-controlled drug delivery systems

In the realm of pharmaceutical science, the controlled release mechanism has emerged as a groundbreaking advancement in drug delivery technology. Unlike traditional immediate-release formulations, which discharge the active compound rapidly into the system, controlled-release systems are designed to dispense medication at a predetermined, sustained rate over an extended period. This strategic approach revolutionizes how drugs are administered and maintained within the body, offering substantial benefits over conventional methods. The essence of controlled release systems lies in their ability to prolong the presence of a drug at its therapeutic target. As illustrated in Figure 5a, these systems ensure that the active compound remains at the desired site for a significantly extended duration after administration. This is achieved through sophisticated formulations that control the rate at which the drug is released into the bloodstream or at the target site, thereby maintaining optimal therapeutic levels for longer periods. This prolonged exposure is critical for treating chronic conditions where maintaining consistent drug levels is essential for efficacy [81]. One of the primary advantages of controlled release mechanisms is the improvement in patient adherence. Medications that require frequent dosing often suffer from poor compliance due to the inconvenience and forgetfulness associated with multiple daily doses. By contrast, controlled-release formulations reduce the frequency of dosing, simplifying the medication regimen and making it easier for patients to stick to their prescribed schedules. This is particularly beneficial in managing chronic diseases, where long-term adherence to medication is crucial for maintaining health and preventing complications. Frequent dosing of medications can lead to peaks and troughs in drug plasma concentrations, often resulting in unwanted side effects. Immediate-release formulations, which deliver a rapid burst of medication, can cause high peaks in plasma levels, leading to an increased risk of adverse reactions. Controlled release systems mitigate this issue by ensuring a more steady and predictable release of the drug, thereby avoiding the sharp spikes in concentration typically associated with the immediate release [82].

This steadier release minimizes the likelihood of side effects and enhances the overall safety profile of the medication. Another significant benefit of controlled release mechanisms is their ability to maintain a more consistent plasma concentration of the drug. Immediate-release formulations can cause rapid increases in drug levels, leading to spikes that can be both therapeutically and toxically significant. In contrast, controlled release systems provide a more gradual and sustained release, resulting in smoother plasma concentration profiles. This not only enhances the therapeutic efficacy of the drug but also reduces the risk of toxicity and other negative outcomes associated with high peak levels [81,82].

### Advanced drug delivery systems: IPN hydrogels

In the pursuit of more effective and tailored drug delivery mechanisms, researchers have developed various systems to control the release of active pharmaceutical ingredients. Among these, IPN hydrogels stand out for their ability to provide sustained and controlled drug release, essential for improving therapeutic outcomes and patient compliance. IPN hydrogels represent a versatile and highly tunable platform for controlled drug delivery. These hydrophilic networks of polymers can absorb substantial amounts of water or biological fluids, leading to their swelling and, consequently, the gradual release of the entrapped drug [81,82]. The swelling capacity and porosity of hydrogels are key attributes that influence their drug-release characteristics. IPN hydrogels can be engineered to encapsulate drugs or other therapeutic agents within their network, allowing for a slow and sustained release as the hydrogel gradually degrades or as the drug diffuses out. This property makes IPN hydrogels particularly suitable for applications requiring prolonged drug release at the target site [81,82]. IPN hydrogels often combine synthetic polymers with natural, biodegradable, and biocompatible polymers to leverage the advantages of both types. Synthetic polymers contribute to the structural integrity and controlled release properties, while natural polymers offer biocompatibility and minimize adverse immune responses. Natural polymers are a popular choice in the development of IPN hydrogels due to their inherent biocompatibility and biodegradability for drug release. Some commonly used natural polymers include:

- Dextran: Known for its ability to form hydrogels with good swelling capacity, it is suitable for drug delivery applications [83].
- Liposomes: They are spherical vesicles composed of one or more phospholipid bilayers, which make them excellent carriers for controlled drug release.
- Guar gum: A natural polysaccharide that forms gels and is used for its thickening and stabilizing properties in controlled-release formulations [84].
- Xanthan gum: Frequently used in combination with other polymers to enhance the gel strength and stability of IPN hydrogels [85].
- Hyaluronic acid: Valued for its biocompatibility and ability to form IPN hydrogels that mimic the ECM, making it ideal for tissue engineering and drug delivery [86,87].
- Chitosan: Derived from chitin, chitosan is widely used for its biodegradability and capacity to form films and IPN gels, providing a conducive environment for sustained drug release [87,88].
- Alginate: Extracted from seaweed, alginate forms IPN hydrogels in the presence of divalent cations like calcium, offering a straightforward and versatile method for encapsulating drugs [89-91].

These natural polymers not only support the structural framework of IPN hydrogels but also enhance their functional capabilities, making them ideal candidates for developing advanced controlled-release drug delivery systems.

### Controlled release mechanisms in IPN hydrogels

IPN hydrogels offer advanced capabilities for controlled drug release through various mechanisms. Understanding these mechanisms is crucial for designing hydrogels that can deliver drugs effectively over extended periods. The stages of this mechanism include:

1. Diffusion-controlled release: In diffusion-controlled release systems, the drug molecules move through the hydrogel matrix driven by concentration gradients. This process is influenced by the size of the drug molecules and the porosity of the IPN hydrogel. The drug diffuses from regions of high concentration within the hydrogel to areas of lower concentration outside it, leading to a gradual release (Figure 5b). The rate of drug release can be fine-tuned by adjusting the pore size and the overall structure of the hydrogel network.
2. Swelling-controlled release: Swelling-controlled release depends on the ability of IPN hydrogel to absorb water or biological fluids, which causes it to expand. This swelling increases the mesh size of the hydrogel, facilitating the release of the encapsulated drug. The degree of swelling and, consequently, the release rate can be influenced by environmental stimuli such as pH, temperature, or ionic strength. The responsiveness of IPN hydrogel to these stimuli makes it possible to design systems that release drugs in a controlled manner when triggered by specific conditions.
3. Degradation-controlled release: IPN hydrogel degradation provides another mechanism for drug release, which can occur through hydrolytic or enzymatic pathways:
  - Hydrolytic degradation: Involves the cleavage of polymer bonds by water, leading to the gradual breakdown of the hydrogel matrix. This method is often utilized in hydrogels composed of synthetic polymers designed to degrade at a predictable rate.
  - Proteolytic degradation: Relies on enzymes to break down the polymer network. This is especially relevant for hydrogels containing natural polymers, which are more susceptible to enzymatic action.
  - Degradation-controlled systems are particularly advantageous for applications where a long-term, sustained release is desired, as the drug is released as the hydrogel matrix itself disintegrates over time.

Tailoring release kinetics through IPN hydrogel design: The release kinetics from IPN hydrogels can be precisely controlled by manipulating several key parameters:

- Polymer chain structure: Modifying the chemical structure of the polymers used in the hydrogel can influence its degradation rate and mechanical properties.
- Polymer concentration: Higher polymer concentrations typically lead to denser networks, slowing the release rate.
- Crosslinking density: Increasing the crosslinking density generally results in smaller pore sizes and a more rigid network, reducing the rate of drug diffusion and slowing the overall release.

A notable example of IPN hydrogel application is the study conducted by Puertas Bartolome and colleagues. They synthesized a hydrogel by crosslinking chitosan and oxidized HA, combined with a synthetic catechol terpolymer. This hydrogel was further coordinated with iron to form a robust IPN structure. The resultant hydrogel demonstrated the ability to sustain and control the release of catechol *in situ* for up to 21 days. This was achieved through a combination of diffusion and degradation mechanisms, providing a continuous and stable drug release profile [88,89]. This innovative approach underscores the versatility and potential of IPN hydrogels in developing sophisticated drug delivery systems that can be tailored to meet specific therapeutic needs.

### Advanced controlled release in IPN Hydrogels

Controlled drug release mechanisms in IPN hydrogels are critical for achieving sustained and precise delivery of therapeutic agents. These mechanisms, influenced by various physicochemical properties and environmental factors, determine how effectively the drug is released over time. Higher concentrations create denser networks with smaller pore sizes, slowing diffusion. Conversely, lower polymer concentrations lead to more porous structures, facilitating faster drug release. The extent to which the liquid medium penetrates the polymer matrix can affect the diffusion rate. This penetration depends on the hydrophilicity of the polymer and its affinity for the surrounding medium [90-92]. Attributes such as molecular weight polymers, crosslinking density, and hydrophilic or hydrophobic balance play crucial roles in defining the diffusion characteristics of drugs. The type of polymers and their ratio in the hydrogel matrix dictate the degree of swelling. For example, IPN hydrogels containing more hydrophilic components tend to swell more in aqueous environments. Combining synthetic and natural polymers to form IPN hydrogels can yield materials with superior mechanical strength, stability, and functionality.

An illustrative example of the complexities involved in controlled release systems is provided by E. Bulut [90], who studied the release dynamics of ibuprofen from ionically crosslinked IPN beads. These beads were composed of sodium alginate and methylcellulose, crosslinked with ferric chloride (FeCl_3_). Several parameters influenced the drug release from these beads: i) ibuprofen/polymer ratio: Increasing the ratio led to higher drug loading but reduced the release rate, as a denser polymer matrix was formed around the drug; ii) crosslinking time: Longer crosslinking times resulted in more tightly bound networks, slowing down the drug release; iii) FeCl_3_ concentration: Higher concentrations of FeCl_3_ created stronger ionic crosslinks, further restricting the diffusion of ibuprofen, and iv) sodium alginate content: As the sodium alginate content increased, the network density also increased, reducing the diffusion of drug rate. The study demonstrated that optimal drug entrapment efficiency was achieved at an ibuprofen/polymer ratio of 1/4, balancing drug loading and release kinetics [90]. This highlights the need for precise control over the formulation parameters to tailor the release profile for specific therapeutic applications. K. Sellamuthu [92] formulated IPN hydrogel beads containing the antidiabetic drug glipizide through encapsulation within sodium alginate and xanthan gum biopolymers using the ionotropic gelation method with calcium chloride as the crosslinking agent. *In vitro* dissolution assessments revealed that the release of the drug is influenced by the ratio of alginate-xanthan gum, with the 7:3 ratio demonstrating increased release after 9 hours [92]. Polymers can exhibit sensitivity to various environmental stimuli such as pH [93-96], temperature [97], ionic strength [98], solvents [99], and more. These stimuli have the potential to alter the swelling, diffusion, or ionic interactions within the matrix, consequently affecting the controlled release of active substances by either decreasing or increasing them (Table 4).

**Table 4.** Stimuli in the medium affecting the swelling of IPN hydrogels for advanced drug release applications.

Stimuli	Mechanism	Ref.
pH	Under acidic conditions, acidic groups undergo protonation, resulting in polymer chain repulsion and heightened swelling. Conversely, under basic conditions, basic groups undergo protonation, leading to diminished swelling due to charge neutralization and subsequent chain collapse.	[93-96]
Temperature	Hydrogels demonstrate swelling characteristics that vary with temperature alterations. An instance of a polymer responsive to temperature is poly(N-isopropylacrylamide) (PNIPAAm).	[97]
Ionic strength	It is determined by the concentration of ions within the medium. Elevated ionic strength shields charge on polymer chains, diminishing electrostatic repulsion and encouraging chain collapse, thereby inducing swelling. Conversely, reduced ionic strength weakens this shielding effect, permitting greater swelling as a result of heightened electrostatic repulsion between charged polymer chains.	[98]
Solvents	Alterations in solvent polarity, viscosity, or dielectric constant can influence interactions between polymers and solvents, as well as the mobility of polymer chains.	[99]

Several researchers have investigated the impact of pH variations on IPN hydrogel swelling and its correlation with drug release. For instance, Y. Shin [93] and colleagues fabricated IPN hydrogels composed of bacterial succinoglycan and carboxymethylcellulose (SG/CMC) polymers using ionic crosslinking between carboxyl and Fe^3+^ groups. They loaded the drug 5-fluorouracil into these hydrogels to examine its release behaviour. Their findings revealed that the hydrogel exhibited pH-responsive behaviour, resulting in matrices with enhanced mechanical strength and swelling capacity. Moreover, they observed a significant increase in the release of 5-fluorouracil drug at physiological pH (pH 7.4) compared to acidic pH (pH 1.2) [93]. In a separate investigation conducted byS. Rai [94], an IPN hydrogel comprising areca cellulose and guar gum grafted with poly(N,N'-dimethylacrylamide) was synthesized using a microwave irradiation technique. N,N-methylenebisacrylamide (MBA) served as the crosslinking agent. The resulting IPN gel was loaded with metformin hydrochloride, an antidiabetic drug. The findings demonstrated full drug release within 6 hours at pH 1.2 and 10 hours at pH 7.4 [94].

In contrast, A. Biswas [95] developed IPN hydrogel beads using polyvinyl alcohol and hydrophilic polysaccharides like xanthan gum, guar gum, gellan gum, and sodium alginate. These beads aimed to enhance the solubility of diclofenac sodium and facilitate its targeted delivery through ionic gelation. The findings revealed that the diclofenac-loaded beads demonstrated a sustained drug release profile dependent on pH [95]. In a separate study, B. Farasati Far *et al.* [96] developed two multireactive biohydrogels comprising chitosan-graft-glycerol (CS-g-gly) and carboxymethyl chitosan-graft-glycerol (CMCS-g-gly), which encapsulated the chemotherapeutic drug vincristine sulphate. These hydrogels exhibited considerable swelling capacities in acidic environments. The hydrophobic chemotherapeutic vincristine was effectively encapsulated and released from the nanohydrogel. *In vitro* release assessments indicated that CS-g-gly and CMCS-g-gly hydrogels enabled sustained and pH-responsive release, particularly under slightly acidic conditions (pH 5) for 120 hours [96]. Furthermore, G. R. Mahdavinia *et al.* [89] explored the impact of nanoparticle-incorporated nanocomposite IPN hydrogels composed of kappa-carrageenan, PVA, and magnetite loaded with diclofenac sodium. The release profile of diclofenac sodium demonstrated dependence on the hydrogel composition and external stimuli. Maximum drug release occurred under phosphate buffer solution (PBS) conditions, while low drug release was observed at pH 1.2 due to the limited solubility of drug in acidic environments [89].

Temperature represents another variable capable of influencing drug release, as alterations in temperature may impede certain interactions between the polymers and the surrounding medium, consequently affecting hydrogel swelling and, by extension, drug release. In this context, S. M. R. Dadfar *et al.* [97] devised a series of semi-IPN hydrogels comprising temperature-sensitive N-isopropylacrylamide (NIPAA) and pH-sensitive netilmaleamic acid (NEMA) units, along with sodium alginate. These hydrogels were synthesized via free radical polymerization in the presence of MBA as a crosslinker and loaded with the drug doxorubicin hydrochloride. Notably, the incorporation of pH-sensitive polymers may influence the sensitivity of temperature-sensitive monomers. Therefore, the findings indicate that the combination of these polymers yields an IPN hydrogel responsive to both stimuli, with no restrictions on the NEMA content, and featuring a gradual drug release under simulated physiological conditions [97]. Another critical factor impacting drug release is ionic strength, as demonstrated by B. Wang *et al.* [98], who investigated ionic strength-sensitive hydrogels containing succinic anhydride-modified xanthan gum loaded with gentamicin. The sustained release of gentamicin was observed to be responsive to changes in ionic strength, persisting for 9 days under physiological conditions [98]. The ionic strength can alter the ionization of the drug and also affect the swelling of the hydrogel, which may create solvent-polymer interactions that can affect the diffusion of the drug into the surrounding medium. IPN hydrogels represent versatile platforms for controlled drug release with significant potential to improve the safety and efficacy of pharmaceutical treatments. Continued innovation is essential for advancing IPN hydrogel-based drug delivery systems and translating them into clinical applications for the benefit of patients.

## IPN hydrogels as bioinks for 3D bioprinting

In the realm of 3D bioprinting, the choice of bioink is pivotal for creating functional and structurally sound biological constructs. IPN hydrogel-based bioinks have become a cornerstone in this field due to their ability to mimic the natural ECM environment. Here, we explore the diverse categories of IPN hydrogels used in bioink formulations and their unique characteristics and applications [100,101].

### Classification of hydrogels for bioinks

Hydrogels can be broadly classified into several categories based on their composition and structural properties:

- Conventional hydrogels: Conventional hydrogels consist of a single polymer network. They are typically formed through covalent or non-covalent interactions facilitated by the functional groups of polymer or segments along its chain. These hydrogels are straightforward but can be limited by their mechanical properties and lack of multifunctionality [100,101].
- IPN hydrogels: IPN hydrogels combine two distinct polymer networks, where one network is often stiff and brittle, while the other is soft and ductile. This configuration imparts superior toughness and fracture resistance compared to conventional hydrogels. IPNs can be classified into semi-IPNs, where only one network is crosslinked, and full-IPNs, where both networks are crosslinked. The interplay of reversible covalent and non-covalent interactions enhances their mechanical robustness and functionality for printing [100,101].
- Nanocomposite hydrogels: Nanocomposite hydrogels are reinforced with nanoparticles such as carbon nanotubes, graphene, silica, and metal oxides, which are embedded into the hydrogel matrix. These nanoparticles significantly improve the mechanical strength and toughness of the hydrogels, making them suitable for demanding applications in tissue engineering and 3D bioprinting. The organic-inorganic nature of these hydrogels enables them to integrate with cellular environments effectively, promoting cell viability and function [100,101].
- Supramolecular hydrogel inks: Supramolecular hydrogels are designed with highly tunable surface structures that enhance cell-scaffold interactions, which are critical for tissue engineering applications. These hydrogels leverage non-covalent interactions such as hydrogen bonding, π-π interactions, and host-guest chemistry to form reversible and dynamic networks. Their customizable properties facilitate improved mechanical strength and printability, essential for creating intricate 3D constructs [100,101].
- Multimaterial inks: Multimaterial inks differ from IPN hydrogels in that they integrate multiple materials and cell types within a single construct, often without relying solely on chemical crosslinking. This approach allows for the incorporation of various hydrogel precursors and cellular components, providing versatile options for creating complex tissue models. These inks are particularly valuable for applications requiring distinct material properties or gradients within a single printed structure [100,101].

### Bioinks

Bioinks are specialized materials designed to encapsulate cells and provide a supportive ECM-like environment during bioprinting. They must maintain cell viability and function under the mechanical stresses of printing [102]. Different formulations of bioinks have been used:

- Alginate-based bioinks: Alginate is a naturally occurring polymer derived from brown seaweed. It forms IPN hydrogels upon exposure to divalent cations like calcium. However, alginate lacks intrinsic cell-binding sites, which limits its ability to support cell adhesion and proliferation. To address this, alginate can be chemically modified with peptides or blended with high molecular weight polymers such as cellulose to enhance its biofunctionality [102,103].
- Gelatin-based bioinks: Gelatin, derived from collagen, is rich in cell-binding RGD domains, promoting cell adhesion. At physiological temperature, pure gelatin becomes a low-viscosity solution, posing challenges for bioprinting. Blending gelatin with more viscous polymers like hyaluronic acid, alginate, or polyethylene glycol (PEG) improves its printability and structural integrity in 3D constructs [103,104].
- Collagen-based bioinks: Collagen is the most abundant protein in the ECM and provides excellent support for cell attachment and growth. However, its high viscosity can hinder bioprinting, and its mechanical strength is relatively weak. To overcome these limitations, collagen is often blended with other polymers such as agarose, chitosan, or hyaluronic acid to enhance its printability and structural fidelity [103,104].
- Fibrin-based bioinks: Fibrin forms through the polymerization of fibrinogen and thrombin, creating a natural matrix that supports cell adhesion. Its low viscosity makes it suitable for inkjet bioprinting, but rapid and irreversible gelation at body temperature can complicate the printing process. This necessitates careful control of the bioprinting environment to maintain fibrin's printability [103,104].
- HA-based bioinks: HA is a highly viscous, naturally occurring polymer that supports cell growth and proliferation. Its viscosity is concentration-dependent, which influences its printability and mechanical strength. To improve these properties, HA is often crosslinked or blended with other materials to enhance its structural fidelity and resistance to hydrolysis [103,104].
- Decellularized ECM (dECM): dECM is derived from animal tissues and retains many of the native biological cues of ECM. It provides a rich environment for cell growth and differentiation but often has low viscosity, leading to poor printability. To enhance its utility in bioprinting, dECM is frequently blended with other polymers or crosslinked to improve its mechanical properties [103,104].
- Silk-based bioinks: Silk proteins, produced by silkworms and spiders, offer high molecular weight and excellent mechanical properties. They are biocompatible, degrade slowly, and protect cells from stress during printing. However, silk-based bioinks can cause nozzle clogging due to their high viscosity and lack cell binding domains, which limits their application to certain cell types [103,104].
- Chitosan-based bioinks: Chitosan is a polysaccharide with inherent antibacterial properties, making it useful for tissue engineering applications. It forms viscous hydrogels suitable for bioprinting but requires blending with other polymers like agarose or alginate to improve structural integrity and printability. Rapid dissociation of chitosan in physiological conditions and lack of cell binding domains restrict its use to specific applications for 3D printing [103,104].
- PEG-based bioinks: PEG is a versatile, hydrophilic polymer that supports nutrient diffusion and cell growth. It is often used to enhance the mechanical properties of bioinks through crosslinking. Biocompatibility of PEG and customizable mechanical properties make it a popular choice for bioink formulations, although it does not inherently support cell binding [103,104].
- Pluronic acid-based bioinks: Pluronic acid, a triblock copolymer, is known for its thermosensitive gelation properties and high resolution in printed constructs. While it supports high-quality printing, its weak mechanical integrity and poor cell support limit its application in more demanding bioprinting scenarios. Pluronic bioinks are often used in combination with other materials to overcome these challenges [103,104].

### Key features of bioinks

**Figure 6.**
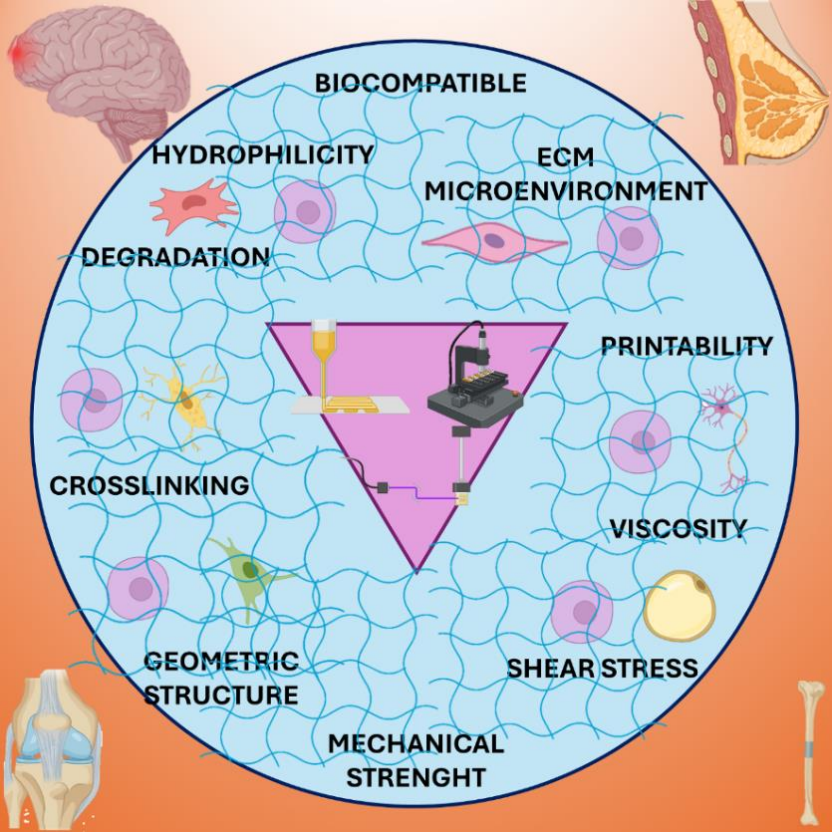
Ideal parameters of bioinks based on IPN hydrogels for 3D bioprinting

Figure 6 presents the ideal parameters necessary for the successful performance of an IPN hydrogel-based bioink. For successful bioprinting, bioinks must possess several critical properties:

- Biocompatibility: Bioinks must be safe for encapsulated cells, ensuring no toxic, allergic, or adverse immune responses. This extends to the materials used, their degradation products, and the bioprinting process [100].
- Ideal ECM microenvironment: The bioink should mimic the ECM, providing appropriate pore volume, shape, and interconnectivity to facilitate cell growth and nutrient diffusion. Incorporating biological cues, like RGD sequences, can enhance cellular proliferation and function within the printed structure [104,105].
- Hydrophilicity: Bioinks should have moderate hydrophilicity, with water contact angles less than 90°, to support cell attachment. Adjusting the hydrophilicity through functional groups can improve cell affinity and integration with the hydrogel [104].
- Degradation mechanism: The degradation rate of bioinks should match the rate of tissue regeneration, releasing non-toxic byproducts. Tailoring the degradation properties through chemical modifications or cell-responsive sites ensures a balance between stability and gradual resorption [104].
- Viscosity and shear stress: Bioinks should exhibit shear-thinning behaviour to facilitate extrusion and prevent nozzle clogging during printing. The viscosity should be optimized to support printability while maintaining the mechanical stability of the printed structure [104,105].
- Mechanical strength: Bioinks need sufficient mechanical strength to maintain the structural integrity of printed constructs. This can be achieved by adding reinforcing materials or adjusting the bioink composition to match the mechanical properties of the target tissue [105].
- Crosslinking: Crosslinking of IPN hydrogels in bioinks is crucial for achieving the desired viscoelastic properties and ensuring quick gelation post-printing. This process is typically achieved under physiological conditions through chemical, physical, or enzymatic methods. Chemical crosslinking involves the use of crosslinking agents that react with functional groups within the hydrogel, providing stability and mechanical integrity to the printed structure. Physical crosslinking utilizes interactions like hydrogen bonding or electrostatic forces, offering reversibility and tunability to the crosslinked network. Enzymatic crosslinking, on the other hand, utilizes enzymes to catalyse reactions that form crosslinks, which is advantageous for cell-laden bioinks where cytocompatibility is paramount [104,105].
- Printability: Printability refers to the ability of bioinks to maintain shape integrity and fidelity during the bioprinting process. Several factors influence printability, including bioink concentration, hydrophilicity, surface tension, self-crosslinking ability, cell density, and properties of the printer nozzle. The shear-thinning behaviour of bioinks allows them to flow under pressure during extrusion-based printing, facilitating deposition and reducing the risk of nozzle clogging. To solidify printed layers, bioinks can be crosslinked using methods such as UV irradiation after each layer deposition, ensuring structural stability without compromising cell viability [100-105].
- Geometric structure: The geometric structure of bioprinted constructs plays a critical role in their functionnality and compatibility with target tissues. For scaffolds intended for cell loading post-printing, optimal spacing between fibres (200-400 μm) promotes cell growth and migration within the hydrogel matrix. In contrast, for hard tissue implants, combining hydrogels with synthetic structures like polycaprolactone (PCL) supports the structural integrity needed for long-term stability and tissue integration [100-105].

### Printing techniques

Efficient bioprinting relies on precise control of printing parameters tailored to specific cell lines and bioink compositions [102]. Various bioprinting approaches have been developed, each offering unique advantages and challenges:

- Extrusion-based bioprinting: Extrusion-based bioprinting utilizes mechanical pressure to extrude bioinks through a syringe nozzle in a controlled manner. This method supports a wide range of bioink viscosities and compositions, making it versatile for fabricating complex tissue structures with high accuracy. Optimizing nozzle diameter is critical to balance mechanical strength with cell viability, ensuring minimal damage during extrusion [101,104,106,107].
- Inkjet-based bioprinting: Inkjet-based bioprinting achieves high printing resolution (b50-300 μm) by ejecting bioink droplets through small nozzles. However, it requires careful control of bioink viscosity (typically <10 cP) and cell concentration (<5×10^6^ cells/ml) to prevent nozzle clogging. Despite its challenges, inkjet bioprinting is valued for its ability to create precise, cell-laden patterns suitable for tissue modelling and drug testing [101,104 106].
- Laser-assisted bioprinting: Laser-assisted bioprinting is a nozzle-free method where bioinks are deposited onto a substrate using laser pulses. This technique supports a wide range of bioink viscosities (1-300 mPa/s) and high cell concentrations (up to 1 × 10^8^ cells/ml) without nozzle clogging. The printhead setup comprises a ribbon, typically a laser-transparent material made up of either glass slide or quartz. A laser-absorbing media (such as Ag, Au, Ti, nanotubes, graphene and TiO_2_) is coated at the donor side of the ribbon and the cell-encapsulated hydrogels are sprayed on the laser-absorbing coating. By controlling laser intensity and pulse duration, precise cell patterning and spatial organization can be achieved, albeit with concerns about residual metallic particles from the laser-absorbing layer [101,104,106-108].

### Applications of bioinks based on IPN hydrogels in regenerative medicine

Bioinks based on IPN hydrogels, comprising biomaterials capable of supporting cell growth and tissue regeneration, are pivotal in tissue engineering. They enable the development of complex 3D constructs with tailored properties essential for various regenerative medicine applications [101,107].

The most recent applications of bioinks based on IPN hydrogels in regenerative medicine are summarized in Table 5. Herein, we explore the diverse applications of bioinks across different tissues and organs:

**Table 5.** Applications and bioinks formulations based on IPN hydrogels in regenerative medicine

IPN bioink	Application	Main characteristics	Ref.
Photo-crosslinkable gelatin	Adipose tissue for breast	Mesenchymal Strong Cells (MSC) differentiated to adipose cell lineage. A large pore size promotes cell distribution and differentiation.	[109]
Photo-crosslinkable gelatin and k-carrageenan	Adipose tissue for breast	Hydrogel exhibits mechanical properties comparable to those of native breast tissue. Cell viability (N 90%) and proliferation for adipose tissue-derived stem cells upon seeding onto scaffolds.	[110]
Alginate	Articular cartilage	Molecular weight of alginate determines its viscosity. Mechanical properties is determined by crosslinking.	[111]
Blend of hyaluronic acid and alginate	Articular cartilage	Optimal mechanical properties. Increase in the expression of chondrogenic gene markers.	[112]
IPN of PEG and alginate	Cartilage like tissue	Human MSC embedded with thrombin Fibrin simulates cartilage’s pericellular matrix and allow a fast diffusion of nutrients.	[113]
IPN Alginate and gelatin methacryloyl (GelMA)	Articular cartilage	Bioprinted construct reinforce with fibers of polycaprolactone. It supports robust chondrogenesis, with encapsulated cells producing hyaline-like cartilage.	[114]
Photo-crosslinkable polyethylene glycol diacrylate (PEGDA), gelatin methacryloyl (GelMA), and chondroitin sulphate methacrylate (CSMA)	Cartilage repair and regeneration *in vivo*	Promote chondrogenic differentiation.	[115]
Photo-crosslinkable supramolecular hydrogel composed of cleavable poly(*N*-acryloyl 2-glycine) (PACG) and methacrylated gelatin (GelMA) (PACG-GelMA)	Osteochondral regeneration	Cell attachment and spreading. Enhancing gene expression of chondrogenic and osteogenic differentiation of human bone marrow stem cells. Around 12 weeks after *in vivo* implantation, the hydrogel scaffold facilitates concurrent regeneration of cartilage and subchondral bone in a rat model.	[116]
Nanocelluse/Chitosan	Osteogenic cell differentiation	Adding cellulose nanocrystals and cells significantly improved the viscosity and mechanical properties. The bioinks were printable without compromising cell viability. -Cellulose nanocrystals promoted osteogenesis of MC3T3-E1 cells in chitosan.	[117]
Alginate crosslinked with calcium ions	Potential vascularization and stem cell migration	Platelet-rich plasma as a source of growth factors. Patient-specific bioink	[118]
Double network: Photo-crosslinkable polyacrylamide (PAAM) and polyurethane (PU) and chemical cross-linked gelatin (Gel)	Bone regeneration	Good mechanical properties. The scaffold is nontoxic and biocompatible. -Desirable osteogenic capability	[119]
Multimaterial bioink: Collagen-elastin-gelatin methacryloyl	Potential neurogeneration	-Antimicrobial properties with genipine used as crosslinker. Potential neural treatments. Stiffness of around 600 Pa	[120]
Alginate/gelatin IPN	Potential bioartificial organ manufacturing	Cytocompatible hydrogels evaluated through *in vitro* 3D cell cultures and bioprinting.	[121]

- Bone regeneration: Bioinks facilitate the fabrication of scaffolds mimicking the hierarchical structure of bone. They promote osteogenic differentiation and integration with existing bone tissue. Applications include bone defect repair and reconstruction.
- Cartilage regeneration: Bioinks support the chondrogenic differentiation of stem cells. They create constructs with suitable mechanical properties for cartilage tissue. Used in treating osteoarthritis and cartilage defects.
- Spinal cord repair: Bioinks aid in the creation of 3D neural constructs. They provide guidance cues for neuronal regeneration. Targeted for spinal cord injuries to restore function.
- Skeletal muscle tissue engineering: Bioinks promote myogenesis and vascularization within muscle constructs. Used for muscle repair after trauma or degenerative diseases.
- Skin regeneration: Bioinks facilitate the formation of skin-like constructs with appropriate barrier functions. Applied in wound healing, burn treatment, and skin grafting.
- Vascular tissue engineering: Bioinks enable the bioprinting of vascular networks within tissue constructs. Used to promote angiogenesis and improve oxygen and nutrient delivery in engineered tissues.
- Ovary and testicular tissue regeneration: Bioinks are employed to create structures that mimic ovarian and testicular microenvironments. Aimed at restoring fertility and hormone production.
- Heart tissue engineering: Bioinks support the bioprinting of cardiac patches with contractile properties. Used for myocardial infarction repair and improving heart function.
- Islet transplantation: Bioinks facilitate the encapsulation and transplantation of pancreatic islet cells. Aimed at providing a functional cure for diabetes.

## Challenges, costs, and complications of IPN hydrogels compared to conventional materials in biological applications

As IPN hydrogels gain traction in various biological applications, it is essential for researchers to understand the critical challenges, costs, and complications associated with their use. This section offers a detailed evaluation of these factors, focusing on key areas such as tissue engineering, controlled drug release, 3D bioprinting, and regenerative medicine. Tissue engineering has evolved significantly with the development of various biomaterials that support cell growth and tissue regeneration. Conventional materials such as collagen, alginate, and PLGA have been extensively used due to their biocompatibility and established protocols [23,43]. However, the advent of IPN hydrogels presents new opportunities and challenges. While collagen and alginate offer good biocompatibility, their mechanical properties often fall short compared to the requirements of more demanding tissue engineering applications [44-50]. PLGA provides better mechanical strength but can introduce complications due to its degradation byproducts. IPN hydrogels generally provide superior mechanical properties and mimic the natural extracellular matrix better. However, their complex structure can introduce variability in performance and require more stringent control during synthesis [15-25]. Collagen and PLGA are expensive and involve complex processing, but they benefit from well-established protocols. Alginate is more affordable but may require modifications for specific applications. The cost of IPN hydrogels is higher due to their sophisticated synthesis and the need for specialized equipment. Scaling up production can be challenging, which may limit their accessibility for large-scale applications [15-25,55-57,122]. Each conventional material has its own set of limitations, such as rapid degradation of collagen and acidic byproducts of PLGA. The sensitivity of alginate to environmental conditions can also impact its effectiveness. Although IPN hydrogels offer enhanced performance, they come with complications related to synthesis complexity, the potential for variability, and the need for precise control over environmental factors [122].

Controlled drug release systems are designed to deliver therapeutic agents at a controlled rate, improving treatment outcomes and patient compliance. Conventional materials like PLGA, alginate, and liposomes have been widely used due to their proven efficacy. However, IPN hydrogels, with their advanced network structures, offer new possibilities for drug delivery. PLGA offers tunable drug release profiles but can be affected by acidic degradation products. Alginate provides controlled release through gelation but is sensitive to environmental conditions. Liposomes allow for encapsulation of a broad range of drugs but face stability issues [15-25]. IPN hydrogels provide advanced control over drug release through their complex network structure. However, their synthesis complexity can introduce variability in drug release profiles, requiring careful optimization. Alginate is relatively cost-effective, while PLGA and liposomes incur higher costs due to synthesis and processing requirements. These conventional materials benefit from established production methods and scalability. The higher costs associated with IPN hydrogels are due to their sophisticated synthesis and specialized equipment. Scaling up production can be challenging, limiting their broader application [89-94]. Each conventional material presents specific complications, such as degradation byproducts for PLGA, sensitivity to environmental changes for alginate, and stability issues for liposomes. While IPN hydrogels offer enhanced performance, they introduce complexities related to synthesis and stability, which can affect drug release consistency [96,123].

3D bioprinting has emerged as a transformative technology in tissue engineering, offering the potential to create complex, biomimetic structures for regenerative medicine. Conventional materials such as gelatin, alginate, and collagen have been widely used as bioinks in 3D bioprinting, but recent advancements have introduced IPN hydrogels as innovative alternatives [101-106]. Gelatin and alginate are less mechanically robust and may require modifications to enhance their properties for complex structures. Collagen offers better biocompatibility but needs additional crosslinking to support structural integrity [107-113]. IPN hydrogels generally provide superior mechanical strength and stability, enhancing the ability to create complex and durable structures. However, achieving the right balance in properties and optimizing synthesis conditions can be challenging. Each conventional material presents specific limitations, such as thermal sensitivity for gelatin, ionic sensitivity for alginate, and high cost for collagen [101,110]. These limitations can affect print quality and stability. While offering advanced properties, IPN hydrogels introduce complexities related to synthesis, variability in print quality, and post-printing stability [110-118]. These factors must be carefully managed to ensure consistent performance [101].

Regenerative medicine aims to restore or replace damaged tissues and organs, utilizing various materials to facilitate tissue repair and regeneration. Conventional materials such as collagen, fibrin, and synthetic polymers have long been employed in regenerative applications. Recently, IPN hydrogels have emerged as advanced alternatives, offering unique advantages due to their complex network structures. Collagen and fibrin offer excellent biocompatibility but may lack the necessary mechanical strength for some applications in regenerative medicine. Synthetic polymers provide tunable properties but can face issues with biocompatibility and degradation byproducts. IPN hydrogels generally provide superior mechanical strength and stability, enhancing their suitability for a wide range of regenerative applications. However, achieving the desired balance in properties and optimizing synthesis conditions can be complex. Conventional materials such as fibrin and synthetic polymers are generally more cost-effective and scalable, making them suitable for large-scale applications [49, 61]. Collagen, while effective, incurs higher costs due to extraction and purification processes [13-33]. Each conventional material presents specific complications, such as mechanical limitations for collagen and fibrin and potential inflammatory responses for synthetic polymers. These limitations can affect their effectiveness in regenerative applications [76-78]. While IPN hydrogels offer advanced properties, they introduce complexities related to synthesis, variability in performance, and cost. Addressing these issues requires careful management to ensure consistent and reliable performance [124].

## Perspectives and conclusions

IPN hydrogels are revolutionizing regenerative medicine by offering unparalleled capabilities in tissue engineering, controlled drug delivery, and 3D bioprinting. These hydrogels combine robust mechanical strength, excellent biocompatibility, and adaptable functionality, making them pivotal in developing advanced, patient-specific therapies. As research in this field advances, the innovative use of IPN hydrogels promises to unlock new therapeutic possibilities, bringing us closer to the goal of treating complex diseases and restoring lost tissue functions. This evolution in medical science will significantly improve the quality of life of patients globally, ushering in a new era of medical treatment and care. The future of IPN hydrogels in regenerative medicine is set to be characterized by continuous breakthroughs and advancements. Key areas of future research and development include: i) Smart and bioresponsive systems: the development of IPN hydrogels that can adapt their properties or release therapeutic agents in response to specific environmental or physiological cues. These smart systems have the potential to transform treatment delivery, offering more dynamic and responsive therapeutic options tailored to individual patient needs; ii) Nanotechnology integration: incorporating nanotechnology into IPN hydrogel systems will enhance precision targeting and functionality in both drug delivery and tissue engineering. Embedding nanoparticles within hydrogels could revolutionize how we control mechanical properties and biological interactions at the molecular level, leading to more effective and nuanced medical treatments; iii) Enhanced bioprinting capabilities: advances in IPN hydrogel bioinks will enable the 3D bioprinting of increasingly complex and functional tissues. This includes the creation of multi-material constructs that integrate various types of cells and biomaterials to more accurately replicate the structure and function of natural tissues. Enhancing the printability and structural fidelity of these hydrogels is essential for their widespread clinical application, and finally, iv) Personalized medicine: the customization of IPN hydrogels to meet the specific needs of individual patients will become more achievable with ongoing advancements in materials science and bioprinting technologies. This personalized approach could result in more effective treatments with fewer side effects tailored to each patient’s unique biological environment, thus optimizing therapeutic outcomes. Some limitations and disadvantages of using IPN hydrogels in biological applications are described below: In tissue engineering, IPN hydrogels are often celebrated for their ability to mimic the extracellular matrix (ECM), making them promising candidates for tissue engineering scaffolds. However, their complexity can also be a limitation. While beneficial for mimicking the ECM, the intricate network structure can lead to difficulties in controlling the mechanical properties and degradation rates. This unpredictability can result in scaffolds that either degrade too quickly, before tissue regeneration is complete, or too slowly, impeding the natural tissue remodelling process. Furthermore, the synthesis of IPN hydrogels often involves complex and time-consuming procedures, which can pose challenges for scalability and reproducibility in large-scale applications [122]. For controlled drug release, the ability of IPN hydrogels to modulate drug release rates is a significant advantage, but this same feature can also be a double-edged sword. The release kinetics of drugs from IPN hydrogels are highly dependent on the composition of the network and environmental conditions, which can be difficult to precisely control. Variations in temperature, pH, or ionic strength can lead to unpredictable release profiles, potentially compromising the therapeutic efficacy. Additionally, the dense network structure, while protective for bioactive molecules, may hinder the diffusion of larger therapeutic agents, limiting the types of drugs that can be effectively delivered using this system [123]. For 3D Bioprinting, IPN hydrogels have shown great promise as bioinks for 3D bioprinting due to their ability to create intricate, biomimetic structures. However, their use in this cutting-edge technology is not without challenges. The rheological properties of IPN hydrogels can be difficult to optimize, affecting printability and resolution. Achieving a balance between viscosity and crosslinking is critical, as overly viscous hydrogels can clog the printing nozzles, while insufficiently crosslinked hydrogels may lack the structural integrity required for stable, high-resolution prints. Moreover, the post-printing stability of IPN hydrogels remains a concern, as their complex structure may lead to unpredictable swelling or shrinkage, affecting the fidelity of the printed constructs [101]. In regenerative medicine, the goal of developing biomimetic IPN hydrogels that closely replicate natural biological environments is ambitious yet fraught with limitations. One significant challenge is the integration of these hydrogels with native tissues. While IPN hydrogels can support cell growth and differentiation, their interface with surrounding tissues is often imperfect, leading to issues with biocompatibility and potential immune responses. Additionally, the long-term stability of IPN hydrogels *in vivo* remains uncertain, as the balance between degradation and tissue regeneration must be carefully controlled to avoid adverse effects. The complexity of designing hydrogels that can dynamically interact with the evolving biological environment is another hurdle that limits their widespread application in regenerative medicine [124]. The ongoing innovation and application of IPN hydrogels are set to profoundly impact regenerative medicine. These advancements will pave the way for more sophisticated and effective therapies, transforming the landscape of medical treatments and offering new hope for patients worldwide. Addressing these limitations and disadvantages is crucial for the successful translation of IPN hydrogels from experimental models to clinical practice, resulting in materials with a promising future in various biological applications.
